# Association between body mass index and risk of breast cancer according to breast cancer subtypes: A systematic review and meta-analysis

**DOI:** 10.1016/j.breast.2026.104710

**Published:** 2026-01-27

**Authors:** Chiara Dauccia, Marco Bruzzone, Luca Arecco, Eva Blondeaux, Marianna Sirico, Riccardo Gerosa, Maria Alice Franzoi, Mariana Brandão, Lorenzo Perrone, Paolo Pedrazzoli, Christine Desmedt, Serena Di Cosimo, Claudio Vernieri, Laetitia Collet, Matteo Lambertini, Evandro de Azambuja, Elisa Agostinetto

**Affiliations:** a Université libre de Bruxelles (ULB), Hôpital Universitaire de Bruxelles (H.U.B), Institut Jules Bordet, Bruxelles, Belgium; b Department of Internal Medicine and Medical Therapy, University of Pavia, Pavia, Italy; c U.O. Epidemiologia Clinica, IRCCS Azienda Ospedaliera Metropolitana, Genova, Italy; d Medical Oncology, Breast & GYN Unit, Department of Clinical and Experimental Oncology and Hematology, IRCCS Istituto Romagnolo per lo Studio Dei Tumori (IRST) "Dino Amadori", Meldola, Forlì - Cesena; e Department of Biomedical Sciences, Humanitas University, Pieve Emanuele, Italy; f Cancer Survivorship Group, Université Paris-Saclay, UVSQ, Gustave Roussy, Inserm, CESP, F-94800, Villejuif, France; g Department of Oncology, Fondazione IRCCS Policlinico San Matteo, 27100, Pavia, Italy; h Laboratory for Translational Breast Cancer Research - KU Leuven, Leuven, Belgium; i Fondazione IRCCS Istituto Nazionale dei Tumori, Milan, Italy; j Department of Oncology and Hematology-Oncology, University of Milan, Milan, Italy; k IFOM ETS, the AIRC Institute of Medical Oncology, Milan, Italy; l Department of Medical Oncology, U.O. Clinica di Oncologia Medica, IRCCS Azienda Ospedaliera Metropolitana, Genova, Italy; m Department of Internal Medicine and Medical Specialties (DiMI), School of Medicine, Università degli Studi di Genova, Genova, Italy

**Keywords:** Body mass index, Breast cancer, Obesity, Overweight

## Abstract

**Background:**

Higher body mass index (BMI) is a risk factor for breast cancer (BC) development, but the relationship with BC subtypes in pre- and post-menopausal women remains unclear.

**Methods:**

We performed a systematic search from PubMed, Embase and Cochrane databases until 09/24 (CRD42020206108) for cohort and case-control studies assessing the association between BMI and BC subtypes and/or menopausal status. BC risk in overweight (BMI 25–29.9 kg/m^2^) or obese (BMI≥30 kg/m^2^) subjects was compared to risk in under/normal weight (BMI<25 kg/m^2^). BC subtypes were classified as i) ER-positive (regardless HER2-status) ii) ER-negative (regardless HER2-status) iii) HER2-positive (regardless ER-status); iv) triple-negative BC (TNBC).

**Results:**

Out of 2841 records screened, 33 studies (9 cohort and 24 case-control) including 2,103,181 women were eligible. Obesity was associated with a modestly increased risk of ER-positive BC (pOR 1.13; 95 % CI 1.03–1.24). In postmenopausal women, obesity was associated with a moderate higher risk of ER-positive BC (pOR 1.29; 95 % CI 1.18–1.41), while overweight was associated with an increased risk of ER-positive (pOR 1.14; 95 % CI 1.06–1.22) and HER2-positive BC (pOR 1.13; 95 % CI 1.05–1.22). In premenopausal women, overweight was linked with reduced risk of ER-positive (pOR 0.80 95 % CI 0.71–0.91) but increased risk of ER-negative BC (pOR 1.15 95 % CI 1.05–1.26) and TNBC (pOR 1.30; 95 % CI 1.15–1.47).

**Conclusions:**

Obesity was associated with a modestly higher risk of ER-positive BC, driven by postmenopausal status. Considering potential confounders, in premenopausal women, higher BMI was associated with lower risk of ER-positive BC, and an increased risk of ER-negative BC.

## Highlights

• Obesity is associated with a moderate increased risk of ER-positive BC.
• The risk of BC by BMI differs according to menopausal status
• Postmenopausal obesity shows stronger association with ER-positive BC
• Further studies are needed to assess BMI-related breast cancer risk.

## 1 Introduction

Obesity is a growing global health concern [1]. Between 1990 and 2022, the global age-standardized prevalence of obesity increased from 4.8 % to 18.5 % in women, with approximately 504 million women affected [1]. Body mass index (BMI), calculated as weight in kilograms divided by height (in meters squared), is widely used to identify the presence and severity of the excess of body adiposity [2]. The use of BMI as a measure of body adiposity comes with several limitations, including its inability to evaluate the distribution of body fat and the difference between lean and fat mass, potentially leading to under- or overestimation of obesity [3,4]. Nonetheless, BMI remains widely accepted due to its ease of use, consistency, and applicability in large-scale epidemiological studies [2].

Obesity increases morbidity and mortality and is a major risk factor for many chronic diseases [2]. Among these, it has been directly associated with 13 types of cancer, including breast cancer (BC) [5,6]. BC is a highly heterogeneous disease, including different subtypes characterized by different prevalence, clinical behavior, and prognosis [7]. Despite obesity has been associated with increased BC risk, the association with subtypes seems to be influenced by different factors, including menopausal status. While the excess of adiposity is a well-established risk factors for estrogen receptor (ER)-positive BC in postmenopausal women, evidence in premenopausal women is less clear. Moreover, data on other BC subtypes remain limited and controversial. To date, no systematic analysis has assessed the association between BMI and the risk of developing BC by subtypes and menopausal status.

We conducted a systematic review and meta-analysis aiming at investigating the association between BMI and the risk of developing BC according to subtypes and menopausal status.

## 2 Methods

### 2.1 Search strategy

A comprehensive literature search was performed from PubMed, Embase and Cochrane databases with no date restriction up to September 15, 2024. The full electronic search strategy is available in Supplementary Table 1. Titles and abstracts of the identified studies were independently reviewed by two reviewers (C.D. and L.A.), with any disagreement resolved through discussion with a third author (E.A.). Cross-referencing of relevant studies and review articles was conducted to ensure all pertinent studies were retrieved. Additionally, abstracts from major conferences from the past two years, including the American Society of Clinical Oncology (ASCO) Annual Meeting, European Society for Medical Oncology (ESMO) Congress, ESMO Breast Cancer Congress, and the San Antonio Breast Cancer Symposium (SABCS), were also retrieved to complement the search. Additionally, reference lists of selected studies were manually reviewed to identify further eligible articles. Only articles published in English were included.

This systematic review and meta-analysis was conducted according to Preferred Reporting Items for Systematic reviews and Meta-Analyses (PRISMA) guidelines [8] and was registered in the PROSPERO database with final registration number: CRD4202020610 [9].

### 2.2 Selection criteria and data extraction

Eligible studies had to fulfill the following criteria: (a) cohort or case-control studies that investigated the association between BMI and the risk of developing BC; (b) availability of information on specific BC subtypes (ER status and/or HER2 status); (c) the odds ratio (ORs) for risk of developing BC had to be reported or could be computed from the data reported in the manuscript. Only studies that categorized BMI according to the World Health Organization (WHO) classification i.e., under/normal weight (BMI<25 kg/m^2^), overweight (BMI 25–29.9 kg/m^2^), and obese (BMI≥30 kg/m^2^) were included in the analysis. Studies with less than 10 patients, including case reports and small case series, were excluded. Studies that did not provide ORs or studies from which ORs for specific outcomes could not be computed, were excluded from the analysis. The variables required for the analysis were extracted independently by two authors (C.D. and L.A.) from all included studies. The Newcastle-Ottawa Scale was employed to assess the quality of the data obtained and the risk of bias in each study (Supplementary Table 2) [10].

### 2.3 Study objectives

The primary objective of this meta-analysis was to investigate the association between BMI and the risk of developing BC according to subtypes. BC subtypes were first categorized based on ER status only, as ER status was reported in the greatest majority of the studies, as opposed to HER2 status that was reported only in a minority of the studies. Selection was conducted regardless of progesterone receptor status, since it was inconsistently reported across studies. Hence, we performed an initial analysis distinguishing BC in two main subtypes: ER-positive vs. ER-negative. Then, to add more granularity to our analysis, we included in the classification of BC subtypes also HER2 status; for this analysis, BC subtypes were classified in four (partially overlapping) categories: I) ER-positive BC, regardless of HER2 status; II) ER-negative BC, irrespective of HER2 status; III) HER2-positive BC, regardless of ER status; and IV) triple-negative breast cancer (TNBC), defined as both ER-negative and HER2-negative. All analyses were stratified by menopausal status, which was defined according to the specific criteria adopted in each included study. In postmenopausal women, a subgroup analysis examined the association of hormone replacement therapy (HRT) on the BMI and BC risk. In all studies included in this subgroup, HRT use was based on self-reported current or past exposure.

### 2.4 Statistical analysis

Pooled odds ratios (pORs) and 95 % confidence intervals (CIs) for each BMI category were calculated using the random-effects model with the DerSimonian and Laird method [11]. The heterogeneity across the studies was assessed by using Higgins' I^2^ index [12]. Sensitivity analyses were performed to determine the stability of the pOR estimates by recalculating them after excluding each single individual study. Publication bias was assessed with Egger's test [13]. All reported p-values are two-sided. All statistical analyses were performed using Stata, software version 16.1 (StataCorp LLC, College Station, TX, USA).

## 3 Results

Of the 2,841 records initially identified, 1,815 remained after duplicate removal. After screening, 1,633 records were excluded for the following reasons: from titles (n = 1,104), from abstract (n = 392), and not relevant conference proceedings (n = 137). The remaining 182 records were sought for full-text retrieval and subsequently assessed for eligibility. Of these, 149 records were excluded for not meeting the eligibility criteria, including the following reasons: no available information on BC subtypes (n = 34), no available information on BMI (n = 55), no available ORs or insufficient data to compute ORs (n = 17), and case reports/case series with fewer than 10 patients (n = 10).

**Fig. 1:**
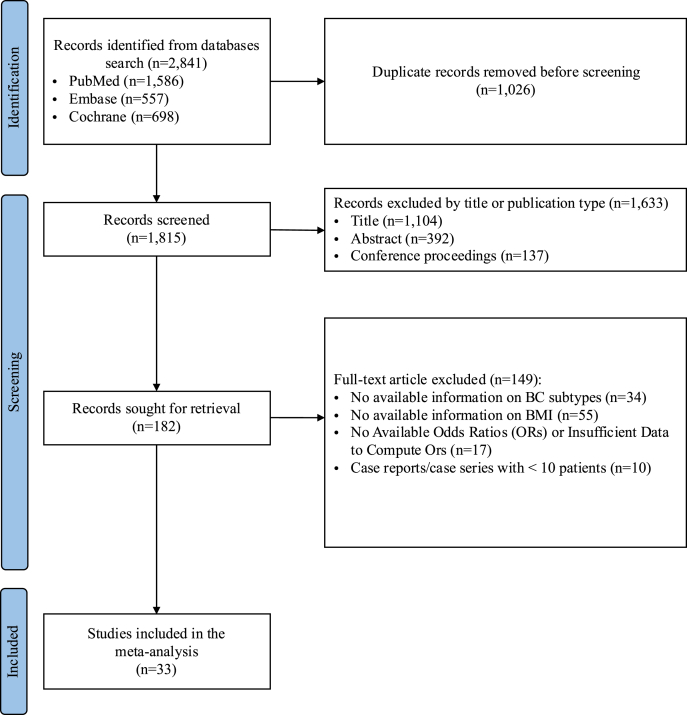
PRISMA diagram of study selection.

Finally, 33 studies evaluating the association between overweight and obesity and the risk of BC according to subtype were considered eligible and included in the present meta-analysis [[14], [15], [16], [17], [18], [19], [20], [21], [22], [23], [24], [25], [26], [27], [28], [29], [30], [31], [32], [33], [34], [35], [36], [37], [38], [39], [40], [41], [42], [43], [44], [45], [46]] (Fig. 1). In Table 1, the main characteristics of each study are presented. Among these, 9 were cohort studies [17,21,24,26,29,31,34,43,45], including 1,964,229 women, and 24 were case-control studies [[14], [15], [16], [17], [18], [19], [20],^22^,^23^,^25^,^27^,^28^,^30^,^32^,^33^,[35], [36], [37], [38], [39], [40], [41], [42],^44^,^46^], including 138,952 women, regardless menopausal status. Some studies included only premenopausal [16,30] or postmenopausal women [14,15,17,[21], [22], [23],^26^,^31^,^33^,^36^,^40^,^45^], but most of studies included both [[18], [19], [20],^24^,^25^,[27], [28], [29],^32^,^34^,^35^,[37], [38], [39],[41], [42], [43], [44],^46^]. Of these studies, 20 were conducted in North America [14,[16], [17], [18], [19], [20], [21],^24^,[27], [28], [29], [30], [31],^33^,^34^,^36^,^38^,^41^,^43^,^44^], 5 in Europe [15,22,23,26,45], 4 in Asia [25,32,35,39], 1 in Latin America [46], 1 study in Oceania [40], 1 in Africa [42], while 1 study was multicontinental [37].

**Table 1:** Main characteristics of the studies included in the meta-analysis (reporting the total number of controls plus cases with known subtype).

First Author	Year	Type of study	Location	Menopausal Status	Age, years	Subtypes	Assessment of weight/height	Study population
Li CI et al. [14]	2006	Case-control	USA	Post-menopausal	65–79	ER+ ER-	Self-reported	1,801
Rosenberg LU et al. [15]	2006	Case-control	Sweden	Post-menopausal	50–74	ER+ ER-	Postal questionnaire	3,651
Ma H et al. [16]	2006	Case-control	USA	Pre-menopausal	20–49	ER+ ER-	Structured questionnaire conducted by qualified stuff.	1,679
Chlebowski RT et al. [17]	2007	Cohort study	USA	Post-menopausal	50–79	ER+ ER-	Self-reported	146,144
Millikan RC et al. [18]	2007	Case-control	USA	Pre- and post-menopausal	20–74	Luminal A Basal-like (TNBC)	In-person interviews by trained nurses.	2,966
Slattery ML et al. [19]	2007	Case-control	USA	Pre- and post-menopausal	25–79	ER+ ER-	Interviewer-administered computerized questionnaire	3,991
Berstard P et al. [20]	2010	Case-control	USA	Pre- and post-menopausal	35–64	ER+ ER-	Interview in person using a structured questionnaire	6,831
Phipps AI et al. [21]	2011	Cohort study	USA	Post-menopausal	50–79	ER+ TNBC	Self-reported height and weight	152,126
Barnes B et al. [22]	2011	Case-control	Germany	Post-menopausal	50–74	ER+ ER-	Face-to-face interviews with trained interviewers.	9,362
Cerne JZ et al. [23]	2012	Case-control	Slovenia	Post-menopausal	50–69	ER+ ER- HER2+ HER2-	Postal questionnaire	1,350
Phipps AI et al. [24]	2012	Cohort study	USA	Pre- and post-menopausal	40–84	ER+ HER2+ (ER-) TNBC	Measured by self-administered questionnaires	554,886
Kawai M et al. [25]	2013	Case-control	Japan	Pre- and post-menopausal	>30	ER+ ER-	Self-administered questionnaire survey	3,559
Horn J et al. [26]	2014	Cohort study	Norway (norwegian women)	Post-menopausal	>55	Luminal A, Luminal B (HER2+/−) HER2+ basal-like (TNBC) Five-negative phenotype (5NP)	Collected through standardized questionnaire carried out by physicians.	19,296
Robinson W et al. [27]	2014	Case-control	USA	Pre- and post-menopausal	20–74	ER+ ER-	Collected by trained nurses	2,610
Bandera EV et al. [28]	2015	Case-control	USA	Pre- and post-menopausal		ER+ ER- TNBC	Medical records and cancer registry data.	12,263
White AJ et al. [29]	2015	Cohort study	USA	Pre- and post-menopausal	35–74	ER+ ER-	Measured during home visits by trained study personnel	253,202
John EM et al. [30]	2015	Case-control	USA	Pre-menopausal	40–59	ER+ ER-	Measured by trained interviewers	2,238
Neuhouser ML et al. [31]	2015	Cohort study	USA	Post-menopausal	50–79	ER+ ER- HER2+ TNBC	Measured at baseline by trained nurses.	62,583
Nagrani R et al. [32]	2016	Case-control	India	Pre- and post-menopausal	20–69	ER+ ER- TNBC	Interview conducted by qualified stuff	2,727
John EM et al. [33]	2016	Case-control	USA	Post-menopausal	35–79	ER+ ER-	Measured by trained interviewers.	5,337
Kerlikowske K et al. [34]	2017	Cohort study	USA	Pre- and post-menopausal	35–74	ER+ ER-	Medical records and cancer registry data	723,451
Wang F et al. [35]	2017	Case-control	China	Pre- and post-menopausal	25–70	ER+ ER-	collected from face-to-face interviews based on a self-designed structured questionnaire.	2,678
McClain KM et al. [36]	2017	Case-control	USA	Post-menopausal	/	Luminal-like (ER/PR+)	Self-reported	1,352
Rudolph A et al. [37]	2018	Case-control	Multicontinental	Pre- and post-menopausal	/	ER+ ER-	Medical Records	5,928
Ma H et al. [38]	2018	Case-control	USA	Pre- and post-menopausal	35–64	Luminal-like HER2+ (ER-/Pgr-) TNBC	collected by trained staff in standardized in-person interviews	5,169
Jeong SH et al. [39]	2019	Case-control	South Korea	Pre- and post-menopausal	35–80	Luminal A Luminal B HER2+ Triple negative	Self-reported	48,570
Dashti GS et al. [40]	2019	Case-control	Australia	Post-menopausal	40–69	ER+	Nurses at dedicated recruitment clinics	1,178
Williams L et al. [41]	2019	Case-control	USA	Pre- and post-menopausal	20–74	Luminal A	Measured by a trained nurse at the time of interview	1,570
Akinyemiju T et al. [43]	2021	Case-control	Nigeria	Pre- and post-menopausal	/	Luminal A Luminal B HER+ TNBC	Obtained by the research nurse at enrolment	455
Friebel-Klingner TM et al. [43]	2021	Cohort study	USA	Pre- and post-menopausal	40–84	Luminal-like TNBC	Medical Records	21,031
Hossain FM et al. [44,45]	2022	Case-control	USA	Pre- and post-menopausal	>20	Luminal A Luminal B HER+ TNBC	From patient records.	11,074
Klintman M et al. [45]	2022	Cohort study	Sweden	Post-menopausal	30–80	Luminal A Luminal B HER+ TNBC	Self-reported	31,510
Gomes K et al. [46]	2022	Case-control	Brazil	Pre- and post-menopausal	>18	Luminal A Luminal B HER+ TNBC	Medical Records	613

### 3.1 Association between BMI and risk of BC in all women (pre and postmenopausal)

**Fig. 2:**
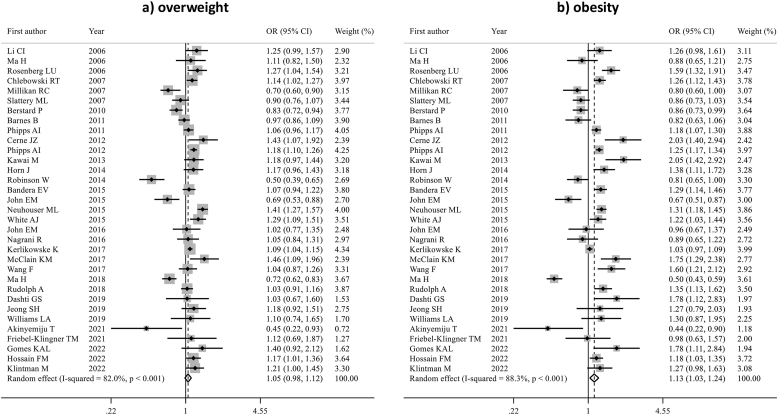
Forest plot displaying the pooled odds ratios for the association between overweight (a) and obesity (b) and the risk of ER-positive breast cancer, compared to underweight/normal weight, in the overall population.

In the overall population, including both pre- and postmenopausal women, obesity was associated with a modestly increased risk of developing ER-positive BC (pOR 1.13, 95 % CI 1.03–1.24, p = 0.008), while this association was not statistically significant for women who were overweight (pOR 1.05; 95 % CI 0.98–1.12) (Fig. 2). There was substantial heterogeneity among studies in both analyses (I^2^ = 82.0 % for overweight and 88.3 % for obesity). No evidence of publication bias was detected (Egger's test: p = 0.303 for overweight, p = 0.940 for obesity).

**Fig. 3:**
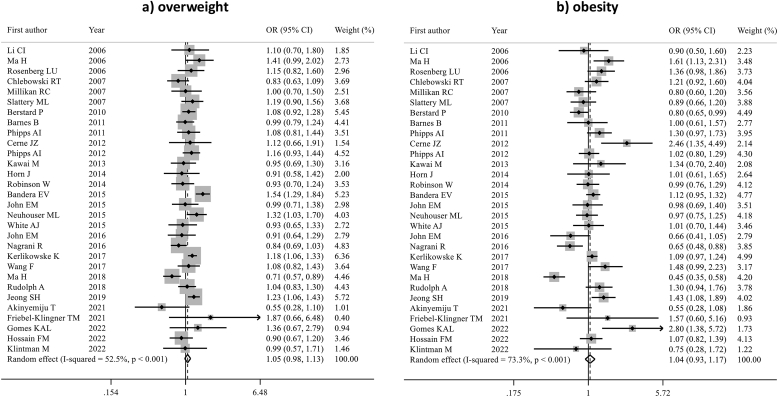
Forest plot displaying the pooled odds ratios for the association between overweight (a) and obesity (b) and the risk of ER-negative breast cancer, compared to underweight/normal weight, in the overall population.

No statistically significant association was found between being overweight or obese and risk of developing ER-negative BC (pOR 1.05; 95 % CI 0.98–1.13 and pOR 1.04; 95 % CI 0.93–1.17, respectively) (Fig. 3). Detailed sensitivity analyses are reported in Supplementary Tables 3–6.

Regarding the HER2-positive BC subtype, 10 studies were included in the analysis (n = 735,506). Women who were overweight were associated with increased risk of HER2-positive BC (pOR 1.09; 95 % CI 1.02–1.16, p = 0.010) while no statistically significant association was found for women with obesity (pOR 1.03; 95 % CI 0.84–1.25) (Supplementary Fig. 1). Detailed sensitivity analyses are presented in Supplementary Tables 7–8.

Regarding TNBC, 13 studies (n = 862,686) were analyzed; no statistically significant association was found between being overweight or obese and the risk of developing TNBC (pOR 1.06; 95 % CI 0.92–1.23 and pOR 1.05; 95 % CI 0.82–1.34, respectively) (Supplementary Fig. 2 and Supplementary Tables 9–10).

### 3.2 Association between BMI and risk of BC in postmenopausal women

**Fig. 4:**
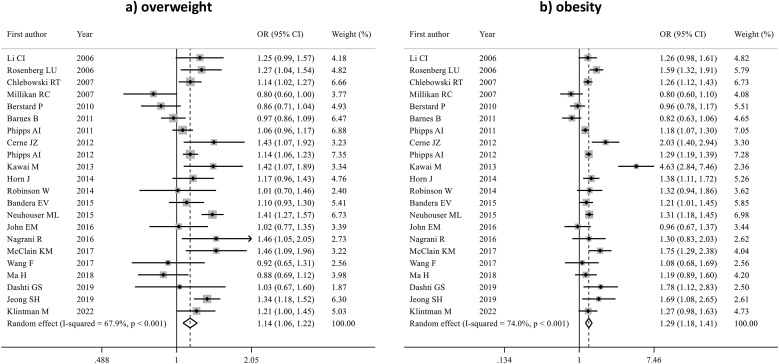
Forest plot displaying the pooled odds ratios for the association between overweight (a) and obesity (b) and the risk of ER-positive breast cancer, compared to underweight/normal weight, in postmenopausal women.

The association between BMI and BC in postmenopausal women was assessed in 22 articles (n = 1,077,949). Overweight/obesity were significantly associated with a moderate increased ER-positive BC risk (pORs 1.14; 95 % CI 1.06–1.22, p < 0.001 for overweight and 1.29; 95 % CI 1.18–1.41, p < 0.001 for obesity) (Fig. 4, Supplementary Tables 11–12). Moderate-to-substantial heterogeneity was found in both analyses (I^2^ = 67.9 % for overweight and 74 % for obesity). No publication bias was detected (Egger's test: p = 0.818 for overweight, p = 0.467 for obesity).

No significant association was observed between overweight/obesity and ER-negative BC risk (pOR 1.04; 95 % CI 0.97–1.11 and pOR 1.02; 95 % CI 0.90–1.16, respectively) (Supplementary Fig. 3, Supplementary Tables 13–14).

Seven studies (n = 723,364) evaluated the association between obesity and overweight and HER2-positive BC in postmenopausal women. Overweight was associated with increased HER2-positive BC risk (pOR 1.13; 95 % CI 1.05–1.22, p = 0.002), but this association was not significant for obesity (pOR 1.19; 95 % CI 0.99–1.43) (Supplementary Fig. 4, Supplementary Tables 15–16)

Nine studies (n = 829,513) evaluated the association between overweight/obesity and TNBC; we observed no statistically significant association (pORs 1.05; 95 % CI 0.88–1.25 and 1.01; 95 % CI 0.72–1.42, respectively) (Supplementary Fig. 5, Supplementary Tables 17–18).

### 3.3 Association between BMI and risk of BC in postmenopausal women according to use of hormone replacement therapy

Four studies (n = 610,426) evaluated the association of BMI and BC risk among postmenopausal women according to HRT use. Among HRT-users, obesity was associated with higher ER-positive BC risk (pOR 1.22; 95 % CI 1.01–1.49, p = 0.047), while this association was not statistically significant for overweight (pOR 1.11; 95 % CI 0.94–1.32). For ER-negative BC, women who were HRT users and overweight showed an increased ER-negative BC risk (pOR 1.17; 1.02–1.35, p = 0.023), while this association was not statistically significant for obesity (pOR 1.10; 95 % CI 0.82–1.46).

Among non-HRT users, obesity was associated with increased ER-positive BC risk (pOR 1.66; 1.17–2.36, p = 0.004) while this association was not significant for overweight (pOR 1.23; 95 % CI 0.98–1.56). Women non-HRT users who were overweight had an increased ER-negative BC risk (pOR 1.21; 95 % CI 1.03–1.42, p = 0.024), while this association was not significant for obesity (pOR 1.11; 95 % CI 0.62–2.01).

### 3.4 Association between BMI and risk of BC in premenopausal women

**Fig. 5:**
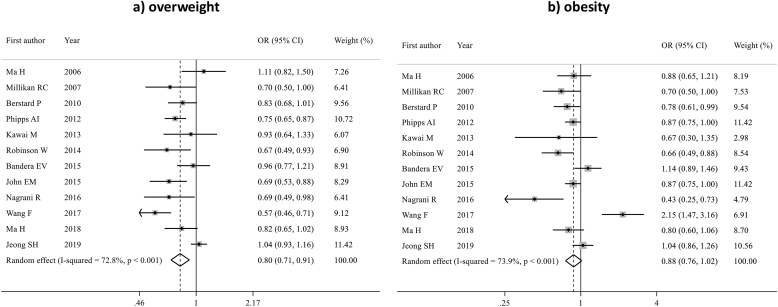
Forest plot displaying the pooled odds ratios for the association between overweight (a) and obesity (b) and the risk of ER-positive breast cancer, compared to underweight/normal weight, in premenopausal women.

Twelve studies, including 646,176 premenopausal women, evaluated the association between overweight and obesity with ER-positive BC. Overweight was associated with lower ER-positive BC risk (pOR 0.80; 95 % CI 0.71–0.91, p = 0.001). A numerically lower risk of ER-positive BC was also observed among women with obesity; however, this association was not statistically significant (pOR 0.88; 95 % CI 0.76–1.02) (Fig. 5). Detailed sensitivity analyses are reported in Supplementary Tables 19–20. There was moderate heterogeneity among studies in both analyses (I^2^ = 72.8 % for overweight and 73.9 % for obesity). No evidence of publication bias was detected (Egger's test: p = 0.148 for overweight, p = 0.714 for obesity).

Premenopausal women who were overweight showed an increased ER-negative BC risk (pOR 1.15; 95 % CI 1.05–1.26, p = 0.002). While a numerically increased risk of ER-negative BC was observed for women with obesity, the association was not significant (pOR 1.11; 95 % CI 0.95–1.29) (Supplementary Fig. 6, Supplementary Tables 21–22)

Three studies (n = 608,625) assessed the association between obesity and overweight and HER2-positive BC. No significant association was found between being overweight or obese and the risk of developing HER2-positive BC (pOR 1.02; 95 % CI 0.91–1.14 and pOR 1.09; 95 % CI 0.89–1.33, respectively) (Supplementary Fig. 7, Supplementary Tables 23–24).

Six studies (n = 626,581) evaluated the association between overweight and obesity and TNBC. Being overweight was associated with an increased risk of TNBC (pOR 1.30; 95 % CI 1.15–1.47, p < 0.001) while this association was not statistically significant for women with obesity (pOR 1.23; 95 % CI 0.95–1.58) (Supplementary Fig. 8, Supplementary Tables 25–26)

## 4 Sensitivity analysis and risk of bias assessment

The sensitivity analysis confirmed the consistency of our findings. This analysis showed that the pooled effect size of the association between BMI and BC risk remained stable with the individual exclusion of each study at a time. Supplementary Table 2 report the quality of data and risk of bias assessment for each study, based on Newcastle-Ottawa Scale. Each study was judged on three broad perspectives: the selection of the study groups; the comparability of the groups; and the ascertainment of the outcome of interest. Sixteen studies received a full score.

## 5 Discussion

In this large meta-analysis, including more than 2 million women, obesity was associated with increased risk of ER-positive BC. Notably, this association was mainly driven by the postmenopausal status, where we observed a stronger association between high BMI and risk of developing ER-positive BC. Conversely, we found in premenopausal women an inverse association between BMI and risk of ER-positive BC, with a reduced ER-positive BC risk among women who were overweight. Overall, the magnitude of the observed associations was moderate.

Higher BMI is an acknowledged risk factor for BC [47]. However, since BC is a biologically and clinically heterogeneous disease, this association may vary across all BC subtypes or patient subgroups, especially by menopausal status. A dose-response meta-analysis previously showed that each 5 kg/m^2^ BMI increase raised BC risk by 2 % [48]. However, that analysis did not account for BC subtypes and/or menopausal status. In our study, in postmenopausal women we found that overweight and obesity were associated with 1.14-fold and 1.29-fold higher ER-positive BC risk, respectively, while we found no significant association for other BC subtypes. Estrogen-driven mechanisms play a key role in affecting the risk of BC [49]. After menopause, the reduction of ovarian estrogens is paralleled by an increased peripheral endogenous estrogens production [50]. Adipose tissue, rich in aromatase that converts androgens into estrogens, represents the primary source of estrogen in postmenopausal women [51]. Excess of adiposity results in a rise in blood estrogen levels, which may contribute to the development of estrogen-driven BC [52]. In addition, obesity-associated insulin resistance, is linked to a reduction in sex hormone-binding globulin (SHBG) [53], which normally limits the bioavailability of sex hormones by binding and sequestering them. SHBG reduction leads to an increase of sex hormone bioavailability, further contributing to increase estrogen levels in the body [54].

In our meta-analysis, obesity was associated with an increased ER-positive BC risk both in HRT users and non-users, suggesting it might represent an independent risk factor for BC development. While data on the interaction between HRT use, obesity, and BC risk are limited, some findings suggest that the contribution of HRT use in increasing BC risk may be less relevant in women with obesity, possibly due to already higher blood estrogen levels [55]. These findings should be interpreted cautiously, as the search strategy was not specifically designed to investigate HRT use.

Consistent to previous studies [48,56], and in contrast to what has been observed in the postmenopausal population, our findings revealed an inverse association between BMI and the risk of developing ER-positive BC in premenopausal women. Specifically, premenopausal women who were overweight had a significant reduction in the risk of ER-positive BC; we also found a trend towards lower risk was also observed in women with obesity, although it did not reach the statistical significance. Before menopause, estrogens are mainly produced by the ovaries under the stimulation of gonadotropins, while the contribution of fat tissue to blood estrogen levels is less relevant compared to what happens in postmenopausal women. Elevated levels of circulating estrogens exert negative feedback on the hypothalamus-pituitary axis, leading to reduced gonadotropin secretion, gonadal stimulation and, ultimately, estrogen production [57,58]. This is the reason why high adiposity may result in irregular and anovulatory menstrual cycles in premenopausal women, thus reducing the exposure of normal epithelial cells in the breast, or BC precursors, to extracellular estrogens [57]. The complex interplay of estrogen pathways and inflammation according to menopausal status may contribute to explain the observed inverse association between BMI and BC risk in premenopausal women [59]. Estrone is the primary estrogen hormone produced after menopause, whereas 17β-estradiol predominates before menopause. These hormones have two opposing effects: while 17β-estradiol exerts anti-inflammatory effects, estrone induces systemic inflammation, potentially contributing to the development and progression of ER-positive BC [59,60].

This meta-analysis showed that being overweight during premenopausal is associated with an increased risk of ER-negative BC (regardless of HER2 status) and TNBC. A trend toward higher risk in premenopausal women with obesity did not reach significance. Estrogen-independent pathways may contribute to BC risk. Excess of adiposity promotes the secretion of pro-inflammatory cytokines and chemokines, leading to a chronic, low-grade inflammation, thus playing a crucial role in promoting BC carcinogenesis [61,62]. Adipokines are produced by the adipose tissue, and they play a crucial role in modulating systemic metabolism, inflammation and insulin sensitivity [63]. In subject with obesity, leptin levels are typically elevated, while adiponectin levels are reduced, and this imbalance may contribute to increased systemic inflammation [64,65]. This inflammation may also worsen systemic insulin resistance, increasing the secretion of insulin and the associated growth factors which have been linked to increase the risk of BC.

In the overall population included in this meta-analysis, especially in postmenopausal women, overweight was associated with an increased risk of HER2-positive BC. HER2-positive BC was classified irrespective of ER status, due to under-reporting in the original studies. This limitation should be considered when interpreting our results. While the impact of BMI on HER2-positive BC risk must be investigated in more detail in future studies, available evidence suggests that the association between high adiposity and survival outcomes may differ according to disease setting [66].

Obesity is a multifactorial condition involving genetic, environmental, psychological and behavioral factors [2,9]. Weight stigma may worsen mental and physical health beyond the metabolic factors associated with obesity, thus potentially worsening unhealthy eating habits and inactivity [2]. Lifelong strategies aiming to support the achievement and maintenance of healthy weight should be actively promoted to reduce BC risk [67]. Although higher BMI seems to be associated with a reduced BC risk in premenopausal women, we would like to highlight the fact that an excess of adiposity before the age of menopause often persists or worsens after menopause, thus becoming a well-known risk factor for the development of BC and other important chronic diseases, including cardiovascular, cerebrovascular and neurological ones. While weight control is not the only preventive measure for BC, we acknowledge its importance as part of a broader strategy for prevention.

Our study has some limitations that should be acknowledged. First, most of included studies were retrospective, with the inherent limitations of this design. Second, significant heterogeneity was observed, reflecting variability in study design and their participant characteristics: not all the studies reported the association between BMI and BC risk according to menopausal status, known to play a major role in this association; most studies considered only the expression of ER to classify tumor subtypes (i.e., ER positive vs. ER negative) while HER2 status was frequently not reported or not stratified according to ER expression, in the context of potential heterogeneous methodologies, and scoring systems across studies, limiting our ability to analyze other BC subtypes; in more than half of the studies, BMI relied on self-reported weight and height, thus implying a potential self-report bias. Additionally, BMI may vary over time, and reliance on a single time-point measurement does not capture longitudinal changes in body weight which may influence the observed associations. While alternative measures of adiposity to BMI may provide a more accurate assessment of fat distribution, their limited availability across studies precluded their inclusion in this meta-analysis. Moreover, this meta-analysis included studies conducted across different countries, thereby capturing heterogeneous populations and healthcare contexts. However, most of the available evidence originated from high-income regions, which may limit the generalizability of our findings to populations from low- and middle-income settings.

On the other hand, our meta-analysis has several strengths: it includes a large cohort of more than 2 million women, thus representing the largest and most up-to-date meta-analysis on this topic so far, providing a comprehensive evaluation of the role of BMI according to not only BC subtypes, but also menopausal status. Our work reports extensive and updated data that are relevant to inform prevention strategies targeting obesity as a risk factor for oncologic and metabolic diseases.

## 6 Conclusion

Obesity was associated with moderate higher ER-positive BC risk. This association appeared to be mainly driven by postmenopausal women. In contrast, among premenopausal women, BMI showed divergent associations by BC subtype, with higher risk observed for ER-negative disease. These results likely reflect the different hormonal status in pre- and postmenopausal women. Maintaining a healthy body weight remains essential throughout life.

## Funding

This study did not receive any funding.

## CRediT authorship contribution statement

**Chiara Dauccia:** Writing – review & editing, Writing – original draft, Conceptualization. **Marco Bruzzone:** Writing – review & editing, Formal analysis. **Luca Arecco:** Writing – review & editing. **Eva Blondeaux:** Writing – review & editing, Methodology, Formal analysis. **Marianna Sirico:** Writing – review & editing. **Riccardo Gerosa:** Writing – review & editing. **Maria Alice Franzoi:** Writing – review & editing, Conceptualization. **Mariana Brandão:** Writing – review & editing, Conceptualization. **Lorenzo Perrone:** Writing – review & editing. **Paolo Pedrazzoli:** Writing – review & editing. **Christine Desmedt:** Writing – review & editing. **Serena Di Cosimo:** Writing – review & editing. **Claudio Vernieri:** Writing – review & editing. **Laetitia Collet:** Writing – review & editing. **Matteo Lambertini:** Writing – review & editing. **Evandro de Azambuja:** Writing – review & editing, Visualization, Supervision. **Elisa Agostinetto:** Writing – review & editing, Validation, Supervision.

## Competing interests

**LA:** travel grant from AstraZeneca. Research funding to his Institution from Gilead.

**EB:** speaker fee from Eli Lilly, research funding (to the institution) from Gilead.

**MAF**: research funding: Resilience (Institution), Speaker honoraria: Novartis.

**LC**: Meeting/travel grants from Astra-Zeneca, GSK, Pharmamar; speaker honoraria from Astra-Zeneca and GSK.

**RG:** Meeting/travel grants from Novartis and Daiichi Sankyo.

**MB**:(all to Institution): Advisory board for Janssen, Sanofi, Pierre-Fabre, Daichii, Pfizer, Boeringher and Amgen. Speaker fee from AstraZeneca, BMS, Janssen, Takeda, MSD, Pfizer. Is/was investigator for AstraZeneca, Boeringher, Merus, Merck, Roche/GNE, Sanofi, iTeos, Pierre-Fabre. Travel grant from Takeda, Sanofi, AstraZeneca, Roche. Consultancy for AstraZeneca.

**SDC:** Given her role as Speciality Editor, Serena Di Cosimo had no involvement in the peer-review of this article and has no access to information regarding its peer-review. Full responsibility for the editorial process for this article was delegated to another journal editor.

**CV**: advisory role for Eli Lilly, Novartis, Astra Zeneca, Pfizer, Daiichi Sankyo, Menarini Stemline; speaker honoraria from Eli Lilly, Novartis, Pfizer, Daiichi Sankyo, Menarini Stemline, MSD, Astra Zeneca, Istituto Gentili, Accademia Nazionale di Medicina; research funding from AIRC, ERC, Ministero della Salute, Roche and Daiichi Sankyo/Astra Zeneca outside the submitted work.

**ML**: advisory role for Roche, Lilly, Novartis, Astrazeneca, Pfizer, Seagen, Gilead, MSD, Exact Sciences, Pierre Fabre, Menarini; speaker honoraria from Roche, Lilly, Novartis, Pfizer, Sandoz, Libbs, Daiichi Sankyo, Takeda, Menarini, AstraZeneca; travel Grants from Gilead, Daiichi Sankyo, Roche; research funding (to the Institution) from Gilead all outside the submitted work.

**EdA**: Financial: Honoraria and/or advisory board from Roche/GNE, Novartis, SeaGen, Zodiac, Libbs, Pierre Fabre, Lilly, Astra-Zeneca, MSD, Gilead Sciences; Travel grants from Astra-Zeneca and Gilead; Research grant to my institution from Roche/GNE, Astra-Zeneca, GSK/Novartis, Gilead Sciences and Seagen/Pfizer; Non-financial: ESMO director of Membership 2023–2025; BSMO President 2023–2026.

**EA**: advisory role and/or honoraria from Eli Lilly, AstraZeneca, Bayer, Abscint, Gilead, Novartis; research grant to her institution from Gilead BeLux; meeting/travel grants from Novartis, Roche, Eli Lilly, Daiichi Sankyo, AstraZeneca, Abscint, Menarini, Gilead (all outside the submitted work).

All remaining authors have declared no conflicts of interest.

## Data availability statement

All data analyzed in this meta-analysis were extracted from previously published, peer-reviewed studies that are publicly available. The datasets supporting the findings of this study can be accessed through the original articles, all of which are cited in the reference list.
