# Supplementary material for: Association between body mass index and risk of breast cancer according to breast cancer subtypes: A systematic review and meta-analysis
Source: Breast. 2026 Jan 27;86:104710. doi: 10.1016/j.breast.2026.104710 (PMC12905738; doi:10.1016/j.breast.2026.104710)
Supplement: Multimedia component 1 [file mmc1.docx]

**Association between Body Mass Index and Risk of Breast Cancer According to Breast Cancer Subtypes:**

**A Systematic Review and Meta-analysis**

**Supplementary material**

Summary

[Supplementary table 1: Search strategy for identification of studies in PubMed. 5](#_Toc201567530)

[Supplementary Table 2: Risk of bias assessment according to The Newcastle-Ottawa Scale (NOS) for Assessing the Quality of non-randomized Studies in Meta-Analysis*. 6](#_Toc201567531)

[Supplementary table 3: Sensitivity analysis for the association between overweight and the risk of ER-positive breast cancer, compared to underweight/normal weight, in the overall population. 10](#_Toc201567532)

[Supplementary table 4: Sensitivity analysis for the association between obesity and the risk of ER-positive breast cancer, compared to underweight/normal weight, in the overall population. 12](#_Toc201567533)

[Supplementary Table 5: Sensitivity analysis for the association between overweight and the risk of ER-negative breast cancer, compared to underweight/normal weight, in the overall population. 14](#_Toc201567534)

[Supplementary Table 6: Sensitivity analysis for the association between obesity and the risk of ER-negative breast cancer, compared to underweight/normal weight, in the overall population. 16](#_Toc201567535)

[Supplementary Figure 1: Forest plot displaying the pooled odds ratios for the association between overweight (a) and obesity (b) and the risk of HER2-positive breast cancer, compared to underweight/normal weight, in the overall population 18](#_Toc201567536)

[Supplementary Table 7: Sensitivity analysis for the association between overweight and the risk of HER2-positive breast cancer, compared to underweight/normal weight, in the overall population 19](#_Toc201567537)

[Supplementary Table 8: Sensitivity analysis for the association between obesity and the risk of HER2-positive breast cancer, compared to underweight/normal weight, in the overall population 20](#_Toc201567538)

[Supplementary Figure 2: Forest plot displaying the pooled odds ratios for the association between overweight (a) and obesity (b) and the risk of TNBC, compared to underweight/normal weight, in the overall population. 21](#_Toc201567539)

[Supplementary Table 9: Sensitivity analysis for the association between overweight and the risk of TNBC, compared to underweight/normal weight, in the overall population 22](#_Toc201567540)

[Supplementary Table 10: Sensitivity analysis for the association between obesity and the risk of TNBC, compared to underweight/normal weight, in the overall population 23](#_Toc201567541)

[Supplementary Table 11: Sensitivity analysis for the association between overweight and the risk of ER-positive breast cancer, compared to underweight/normal weight, in postmenopausal women 24](#_Toc201567542)

[Supplementary Table 12: Sensitivity analysis for the association between obesity and the risk of ER-positive breast cancer, compared to underweight/normal weight, in postmenopausal women 25](#_Toc201567543)

[Supplementary Figure 3: Forest plot displaying the pooled odds ratios for the association between overweight (a) and obesity (b) and the risk of ER-negative breast cancer, compared to underweight/normal weight, in the postmenopausal women 26](#_Toc201567544)

[Supplementary Table 13: Sensitivity analysis for the association between overweight and the risk of ER-negative breast cancer, compared to underweight/normal weight, in postmenopausal women 27](#_Toc201567545)

[Supplementary Table 14: Sensitivity analysis for the association between obesity and the risk of ER-negative breast cancer, compared to underweight/normal weight, in postmenopausal women 28](#_Toc201567546)

[Supplementary Figure 4: Forest plot displaying the pooled odds ratios for the association between overweight (a) and obesity (b) and the risk of HER2-positive BC, compared to underweight/normal weight, in the postmenopausal women 29](#_Toc201567547)

[Supplementary Table 15: Sensitivity analysis for the association between overweight and the risk of HER2-positive breast cancer, compared to underweight/normal weight, in postmenopausal women 30](#_Toc201567548)

[Supplementary Table 16: Sensitivity analysis for the association between obesity and the risk of HER2-positive breast cancer, compared to underweight/normal weight, in postmenopausal women 31](#_Toc201567549)

[Supplementary Figure 5: Forest plot displaying the pooled odds ratios for the association between overweight (a) and obesity (b) and the risk of TNBC, compared to underweight/normal weight, in the postmenopausal women 32](#_Toc201567550)

[Supplementary Table 17: Sensitivity analysis for the association between overweight and the risk of TNBC, compared to underweight/normal weight, in postmenopausal women 33](#_Toc201567551)

[Supplementary Table 18: Sensitivity analysis for the association between obesity and the risk of TNBC, compared to underweight/normal weight, in postmenopausal women 34](#_Toc201567552)

[Supplementary Table 19: Sensitivity analysis for the association between overweight and the ER-positive, compared to underweight/normal weight, in premenopausal women 35](#_Toc201567553)

[Supplementary Table 20: Sensitivity analysis for the association between obesity and the ER-positive, compared to underweight/normal weight, in premenopausal women 36](#_Toc201567554)

[Supplementary Figure 6: Forest plot displaying the pooled odds ratios for the association between overweight (a) and obesity (b) and the risk of ER-negative breast cancer, compared to underweight/normal weight, in the premenopausal women 37](#_Toc201567555)

[Supplementary Table 21: Sensitivity analysis for the association between overweight and the ER-negative, compared to underweight/normal weight, in premenopausal women 38](#_Toc201567556)

[Supplementary Table 22: Sensitivity analysis for the association between obesity and the ER-negative, compared to underweight/normal weight, in premenopausal women 39](#_Toc201567557)

[Supplementary Figure 7: Forest plot displaying the pooled odds ratios for the association between overweight (a) and obesity (b) and the risk of HER2-positive breast cancer, compared to underweight/normal weight, in the premenopausal women 40](#_Toc201567558)

[Supplementary Table 23: Sensitivity analysis for the association between overweight and the risk of HER2-positive, compared to underweight/normal weight, in premenopausal women 41](#_Toc201567559)

[Supplementary Figure 8: Forest plot displaying the pooled odds ratios for the association between overweight (a) and obesity (b) and the risk of TNBC, compared to underweight/normal weight, in the premenopausal women 42](#_Toc201567560)

[Supplementary Table 25: Sensitivity analysis for the association between overweight and the risk of TNBC, compared to underweight/normal weight, in premenopausal women 43](#_Toc201567561)

[Supplementary Table 26: Sensitivity analysis for the association between obesity and the risk of TNBC, compared to underweight/normal weight, in premenopausal women 44](#_Toc201567562)

**Supplementary table 1**: Search strategy for identification of studies in PubMed.

| **Search date** | **Search String*** |
| --- | --- |
| Up to15th September 2024 | ("breast cancer" OR "breast neoplasm" OR “breast tumor” OR “breast tumors” OR “breast tumour” OR “breast tumours” OR "Breast Neoplasms"[MeSH]) AND (“triple-negative” OR “TNBC” OR “ER-positive” OR “luminal” OR “HER2-positive” OR “HER2+” OR “HER2” OR “estrogen receptor” OR “progesterone receptor” OR “hormone receptor” OR “subtype”) AND (“BMI” OR “Body Mass Index” OR “Weight” OR “Height” OR “Obesity” OR “Overweight” OR “Body Size”) AND (“risk” OR “risk factor” OR “etiology”) NOT (animals[MeSH Terms] NOT humans[MeSH Terms]). |

*The following search string was used in PubMed and adapted accordingly for Embase and Cochrane

# **Supplementary Table 2:** Risk of bias assessment according to The Newcastle-Ottawa Scale (NOS) for Assessing the Quality of non-randomized Studies in Meta-Analysis*.

|  | **Selection** | **Comparability** | **Exposure** |
| --- | --- | --- | --- |
| **Jeong SH** | ******** | ****** | ******* |
| **Rudolph** | ******* | ****** | ******* |
| **Kerlikowske K.** | ******** | ****** | ******* |
| **Ma H** | ******** | ****** | ****** |
| **Dashti SG** | ******** | ****** | ******* |
| **Bandera** | ******** | ****** | ******* |
| **William LA** | ******** | ****** | ******* |
| **Wang F** | ******** | ****** | ******* |
| **Horn J** | ******** | ****** | ******* |
| **White AJ** | ******** | ****** | ******* |
| **Nagrani R** | ******** | ****** | ******* |
| **Cerne JZ** | ******* | ****** | ****** |
| **Li CI** | ******* | ****** | ******* |
| **John EM** | ******* | ******* | ******* |
| **John EM** | ******* | ******* | ******* |
| **Phipps A** | ******* | ****** | ******* |
| **Phipps A** | ******* | ****** | ******* |
| **Chlebowski R** | ******* | ****** | ******* |
| **Akinyemiju T** | ******** | ****** | ******* |
| **Hossain FM** | ******** | ****** | ******* |
| **Klintman M.** | ******* | ****** | ******* |
| **Friebel-Klingner TM** | ******** | ****** | ******* |
| **Gomes KAL** | ******** | ****** | ******* |
| **Kawai M** | ******* | ****** | ****** |
| **Robinson WR** | ******** | ****** | ****** |
| **Rosenberg LU** | ******* | ****** | ****** |
| **Berstard P** | ******** | ****** | ****** |
| **Slattery ML** | ******** | ****** | ****** |
| **Setiawan VW** | ******* | ****** | ******* |
| **Canchola AJ** | ******* | ****** | ******* |
| **Millikan RC** | ******** | ****** | ******* |
| **McClain KM** | ******* | ****** | ****** |
| **Neuhouser ML** | ******** | ****** | ******* |
| **Barnes BBE** | ******** | ****** | ****** |
| **Ma H** | ******** | ****** | ****** |

*The Newcastle-Ottawa Scale (NOS) is a tool developed to assess the quality of non-randomized studies included in meta-analysis in order to improve interpretation of meta-analytic results. It consists on a 'star system', in which each study is judged on three broad perspectives: the selection of the study groups; the comparability of the groups; and the ascertainment of either the exposure or outcome of interest for case-control or cohort studies respectively.

# **Supplementary table 3:** Sensitivity analysis for the association between overweight and the risk of ER-positive breast cancer, compared to underweight/normal weight, in the overall population.

| **Study excluded** | **Random effect** | | | **I-squared (%)** | **I-sq. P-value** |
| --- | --- | --- | --- | --- | --- |
|  | **OR** | **95% CI** | **P-value** |  |  |
| Li CI et al 2006 | 1.04 | 0.98-1.12 | 0.212 | 82.4 | <0.001 |
| Ma H et al 2006 | 1.05 | 0.98-1.12 | 0.173 | 82.6 | <0.001 |
| Rosenberg LU et al 2006 | 1.04 | 0.98-1.12 | 0.224 | 82.3 | <0.001 |
| Chlebowski RT et al 2007 | 1.05 | 0.98-1.12 | 0.206 | 82.5 | <0.001 |
| Millikan RC et al 2007 | 1.06 | 1.00-1.14 | 0.062 | 80.6 | <0.001 |
| Slattery ML et al 2007 | 1.06 | 0.99-1.13 | 0.119 | 82.1 | <0.001 |
| Berstard P et al 2010 | 1.06 | 0.99-1.13 | 0.087 | 80.9 | <0.001 |
| Barnes B et al 2011 | 1.05 | 0.98-1.13 | 0.143 | 82.2 | <0.001 |
| Phipps AI et al 2011 | 1.05 | 0.98-1.13 | 0.182 | 82.5 | <0.001 |
| Cerne JZ et al 2012 | 1.04 | 0.97-1.11 | 0.233 | 82.2 | <0.001 |
| Phipps AI et al 2012 | 1.04 | 0.97-1.12 | 0.234 | 81.8 | <0.001 |
| Kawai M et al 2013 | 1.05 | 0.98-1.12 | 0.201 | 82.5 | <0.001 |
| Horn J et al 2014 | 1.05 | 0.98-1.12 | 0.198 | 82.5 | <0.001 |
| Robinson W et al 2014 | 1.07 | 1.01-1.14 | 0.028 | 78.2 | <0.001 |
| Bandera EV et al 2015 | 1.05 | 0.98-1.12 | 0.178 | 82.6 | <0.001 |
| John EM et al 2015 | 1.06 | 0.99-1.13 | 0.073 | 81.3 | <0.001 |
| Neuhouser ML et al 2015 | 1.04 | 0.97-1.11 | 0.274 | 79.6 | <0.001 |
| White AJ et al 2015 | 1.04 | 0.97-1.12 | 0.237 | 82.1 | <0.001 |
| John EM et al 2016 | 1.05 | 0.98-1.12 | 0.156 | 82.5 | <0.001 |
| Nagrani R et al 2016 | 1.05 | 0.98-1.12 | 0.164 | 82.6 | <0.001 |
| Kerlikowske K et al 2017 | 1.05 | 0.97-1.13 | 0.228 | 82.5 | <0.001 |
| McClain KM et al 2017 | 1.04 | 0.97-1.11 | 0.237 | 82.1 | <0.001 |
| Wang F et al 2017 | 1.05 | 0.98-1.12 | 0.163 | 82.5 | <0.001 |
| Ma H et al 2018 | 1.07 | 1.00-1.14 | 0.049 | 78.9 | <0.001 |
| Rudolph A et al 2018 | 1.05 | 0.98-1.13 | 0.165 | 82.5 | <0.001 |
| Dashti GS et al 2019 | 1.05 | 0.98-1.12 | 0.157 | 82.6 | <0.001 |
| Jeong SH et al 2019 | 1.05 | 0.98-1.12 | 0.193 | 82.5 | <0.001 |
| Williams LA et al 2019 | 1.05 | 0.98-1.12 | 0.167 | 82.6 | <0.001 |
| Akinyemiju T et al 2021 | 1.06 | 0.99-1.13 | 0.106 | 82.0 | <0.001 |
| Friebel-Klingner TM et al 2021 | 1.05 | 0.98-1.12 | 0.166 | 82.6 | <0.001 |
| Gomes KAL et al 2022 | 1.05 | 0.98-1.12 | 0.202 | 82.4 | <0.001 |
| Hossain FM et al 2022 | 1.05 | 0.98-1.12 | 0.207 | 82.4 | <0.001 |
| Klintman M et al 2022 | 1.04 | 0.98-1.12 | 0.211 | 82.4 | <0.001 |

Abbreviations: OR, odds ratio; CI, confidence intervals.

# **Supplementary table 4:** Sensitivity analysis for the association between obesity and the risk of ER-positive breast cancer, compared to underweight/normal weight, in the overall population.

| **Study excluded** | **Random effect** | | | **I-squared (%)** | **I-sq. P-value** |
| --- | --- | --- | --- | --- | --- |
|  | **OR** | **95% CI** | **P-value** |  |  |
| Li CI et al 2006 | 1.13 | 1.13-1.24 | 0.012 | 88.6 | <0.001 |
| Ma H et al 2006 | 1.14 | 1.04-1.25 | 0.006 | 88.5 | <0.001 |
| Rosenberg LU et al 2006 | 1.12 | 1.02-1.22 | 0.019 | 88.0 | <0.001 |
| Chlebowski RT et al 2007 | 1.13 | 1.02-1.24 | 0.014 | 88.5 | <0.001 |
| Millikan RC et al 2007 | 1.14 | 1.04-1.25 | 0.005 | 88.3 | <0.001 |
| Slattery ML et al 2007 | 1.14 | 1.04-1.25 | 0.005 | 88.2 | <0.001 |
| Berstard P et al 2010 | 1.14 | 1.04-1.25 | 0.005 | 88.1 | <0.001 |
| Barnes B et al 2011 | 1.14 | 1.04-1.25 | 0.005 | 88.4 | <0.001 |
| Phipps AI et al 2011 | 1.13 | 1.03-1.24 | 0.014 | 88.6 | <0.001 |
| Cerne JZ et al 2012 | 1.11 | 1.02-1.22 | 0.020 | 88.2 | <0.001 |
| Phipps AI et al 2012 | 1.13 | 1.02-1.24 | 0.018 | 88.1 | <0.001 |
| Kawai M et al 2013 | 1.11 | 1.02-1.22 | 0.021 | 88.2 | <0.001 |
| Horn J et al 2014 | 1.12 | 1.02-1.23 | 0.015 | 88.5 | <0.001 |
| Robinson W et al 2014 | 1.14 | 1.04-1.25 | 0.004 | 88.2 | <0.001 |
| Bandera EV et al 2015 | 1.12 | 1.02-1.24 | 0.015 | 88.4 | <0.001 |
| John EM et al 2015 | 1.15 | 1.05-1.26 | 0.003 | 88.0 | <0.001 |
| Neuhouser ML et al 2015 | 1.12 | 1.02-1.23 | 0.016 | 88.2 | <0.001 |
| White AJ et al 2015 | 1.13 | 1.03-1.24 | 0.012 | 88.6 | <0.001 |
| John EM et al 2016 | 1.13 | 1.04-1.24 | 0.007 | 88.6 | <0.001 |
| Nagrani R et al 2016 | 1.14 | 1.04-1.25 | 0.006 | 88.5 | <0.001 |
| Kerlikowske K et al 2017 | 1.14 | 1.03-1.25 | 0.013 | 88.1 | <0.001 |
| McClain KM et al 2017 | 1.12 | 1.02-1.22 | 0.019 | 88.3 | <0.001 |
| Wang F et al 2017 | 1.12 | 1.02-1.23 | 0.017 | 88.4 | <0.001 |
| Ma H et al 2018 | 1.16 | 1.08-1.26 | <0.001 | 81.6 | <0.001 |
| Rudolph A et al 2018 | 1.12 | 1.02-1.23 | 0.015 | 88.5 | <0.001 |
| Dashti GS et al 2019 | 1.12 | 1.02-1.23 | 0.015 | 88.5 | <0.001 |
| Jeong SH et al 2019 | 1.13 | 1.03-1.24 | 0.011 | 88.6 | <0.001 |
| Williams LA et al 2019 | 1.13 | 1.03-1.23 | 0.011 | 88.6 | <0.001 |
| Akinyemiju T et al 2021 | 1.14 | 1.04-1.25 | 0.004 | 88.3 | <0.001 |
| Friebel-Klingner TM et al 2021 | 1.13 | 1.03-1.24 | 0.008 | 88.6 | <0.001 |
| Gomes KAL et al 2022 | 1.12 | 1.02-1.23 | 0.015 | 88.5 | <0.001 |
| Hossain FM et al 2022 | 1.13 | 1.03-1.24 | 0.012 | 88.6 | <0.001 |
| Klintman M et al 2022 | 1.13 | 1.03-1.24 | 0.012 | 88.6 | <0.001 |

Abbreviations: OR, odds ratio; CI, confidence intervals.

# **Supplementary Table 5:** Sensitivity analysis for the association between overweight and the risk of ER-negative breast cancer, compared to underweight/normal weight, in the overall population**.**

| **Study excluded** | **Random effect** | | | **I-squared (%)** | **I-sq. P-value** |
| --- | --- | --- | --- | --- | --- |
|  | **OR** | **95% CI** | **P-value** |  |  |
| Li CI et al 2006 | 1.05 | 0.98-1.13 | 0.194 | 54.1 | <0.001 |
| Ma H et al 2006 | 1.04 | 0.97-1.13 | 0.257 | 52.4 | 0.001 |
| Rosenberg LU et al 2006 | 1.05 | 0.97-1.13 | 0.215 | 54.0 | <0.001 |
| Chlebowski RT et al 2007 | 1.06 | 0.99-1.15 | 0.109 | 51.1 | 0.001 |
| Millikan RC et al 2007 | 1.05 | 0.98-1.14 | 0.177 | 54.0 | <0.001 |
| Slattery ML et al 2007 | 1.05 | 0.97-1.13 | 0.236 | 53.7 | <0.001 |
| Berstard P et al 2010 | 1.05 | 0.97-1.14 | 0.223 | 54.1 | <0.001 |
| Barnes B et al 2011 | 1.06 | 0.98-1.14 | 0.172 | 53.6 | <0.001 |
| Phipps AI et al 2011 | 1.05 | 0.97-1.14 | 0.202 | 54.1 | <0.001 |
| Cerne JZ et al 2012 | 1.05 | 0.98-1.13 | 0.194 | 54.1 | <0.001 |
| Phipps AI et al 2012 | 1.05 | 0.97-1.13 | 0.241 | 53.8 | <0.001 |
| Kawai M et al 2013 | 1.06 | 0.98-1.14 | 0.161 | 53.6 | <0.001 |
| Horn J et al 2014 | 1.06 | 0.98-1.14 | 0.159 | 53.6 | <0.001 |
| Robinson W et al 2014 | 1.06 | 0.98-1.14 | 0.151 | 53.2 | <0.001 |
| Bandera EV et al 2015 | 1.03 | 0.97-1.11 | 0.325 | 37.5 | 0.023 |
| John EM et al 2015 | 1.05 | 0.98-1.14 | 0.173 | 53.9 | <0.001 |
| Neuhouser ML et al 2015 | 1.04 | 0.97-1.12 | 0.277 | 52.1 | 0.001 |
| White AJ et al 2015 | 1.06 | 0.98-1.14 | 0.158 | 53.6 | <0.001 |
| John EM et al 2016 | 1.06 | 0.98-1.14 | 0.151 | 53.4 | <0.001 |
| Nagrani R et al 2016 | 1.07 | 0.99-1.15 | 0.087 | 48.6 | 0.002 |
| Kerlikowske K et al 2017 | 1.04 | 0.97-1.13 | 0.287 | 52.0 | 0.001 |
| Wang F et al 2017 | 1.05 | 0.97-1.14 | 0.203 | 54.1 | <0.001 |
| Ma H et al 2018 | 1.08 | 1.01-1.15 | 0.030 | 39.9 | 0.015 |
| Rudolph A et al 2018 | 1.05 | 0.97-1.14 | 0.194 | 54.0 | <0.001 |
| Jeong SH et al 2019 | 1.04 | 0.97-1.13 | 0.290 | 51.7 | 0.001 |
| Akinyemiju T et al 2021 | 1.06 | 0.99-1.14 | 0.116 | 51.1 | 0.001 |
| Friebel-Klingner TM et al 2021 | 1.05 | 0.98-1.13 | 0.197 | 53.4 | <0.001 |
| Gomes KAL et al 2022 | 1.05 | 0.97-1.13 | 0.203 | 53.8 | <0.001 |
| Hossain FM et al 2022 | 1.06 | 0.98-1.14 | 0.140 | 52.9 | <0.001 |
| Klintman M et al 2022 | 1.05 | 0.98-1.14 | 0.177 | 54.0 | <0.001 |

Abbreviations: OR, odds ratio; CI, confidence intervals.

# **Supplementary Table 6:** Sensitivity analysis for the association between obesity and the risk of ER-negative breast cancer, compared to underweight/normal weight, in the overall population.

| **Study excluded** | **Random effect** | | | **I-squared (%)** | **I-sq. P-value** |
| --- | --- | --- | --- | --- | --- |
|  | **OR** | **95% CI** | **P-value** |  |  |
| Li CI et al 2006 | 1.05 | 0.93-1.17 | 0.456 | 74.1 | <0.001 |
| Ma H et al 2006 | 1.03 | 0.91-1.15 | 0.675 | 72.6 | <0.001 |
| Rosenberg LU et al 2006 | 1.03 | 0.92-1.16 | 0.610 | 73.4 | <0.001 |
| Chlebowski RT et al 2007 | 1.04 | 0.92-1.17 | 0.564 | 73.8 | <0.001 |
| Millikan RC et al 2007 | 1.05 | 0.94-1.18 | 0.396 | 73.7 | <0.001 |
| Slattery ML et al 2007 | 1.05 | 0.93-1.18 | 0.431 | 74.0 | <0.001 |
| Berstard P et al 2010 | 1.06 | 0.94-1.19 | 0.374 | 72.7 | <0.001 |
| Barnes B et al 2011 | 1.04 | 0.93-1.17 | 0.480 | 74.2 | <0.001 |
| Phipps AI et al 2011 | 1.03 | 0.92-1.16 | 0.595 | 73.5 | <0.001 |
| Cerne JZ et al 2012 | 1.02 | 0.91-1.14 | 0.709 | 72.1 | <0.001 |
| Phipps AI et al 2012 | 1.04 | 0.93-1.18 | 0.487 | 74.2 | <0.001 |
| Kawai M et al 2013 | 1.04 | 0.92-1.16 | 0.549 | 74.0 | <0.001 |
| Horn J et al 2014 | 1.04 | 0.93-1.17 | 0.483 | 74.2 | <0.001 |
| Robinson W et al 2014 | 1.04 | 0.93-1.18 | 0.474 | 74.2 | <0.001 |
| Bandera EV et al 2015 | 1.04 | 0.92-1.17 | 0.536 | 73.9 | <0.001 |
| John EM et al 2015 | 1.04 | 0.93-1.18 | 0.471 | 74.2 | <0.001 |
| Neuhouser ML et al 2015 | 1.05 | 0.93-1.18 | 0.465 | 74.1 | <0.001 |
| White AJ et al 2015 | 1.04 | 0.93-1.17 | 0.482 | 74.2 | <0.001 |
| John EM et al 2016 | 1.06 | 0.94-1.18 | 0.362 | 73.3 | <0.001 |
| Nagrani R et al 2016 | 1.06 | 0.95-1.19 | 0.310 | 71.9 | <0.001 |
| Kerlikowske K et al 2017 | 1.04 | 0.92-1.18 | 0.526 | 73.9 | <0.001 |
| Wang F et al 2017 | 1.03 | 0.92-1.16 | 0.622 | 73.4 | <0.001 |
| Ma H et al 2018 | 1.08 | 0.98-1.18 | 0.128 | 57.3 | <0.001 |
| Rudolph A et al 2018 | 1.03 | 0.92-1.16 | 0.590 | 73.6 | <0.001 |
| Jeong SH et al 2019 | 1.03 | 0.92-1.15 | 0.645 | 72.8 | <0.001 |
| Akinyemiju T et al 2021 | 1.05 | 0.94-1.18 | 0.369 | 73.4 | <0.001 |
| Friebel-Klingner TM et al 2021 | 1.04 | 0.93-1.16 | 0.530 | 74.0 | <0.001 |
| Gomes KAL et al 2022 | 1.02 | 0.92-1.14 | 0.689 | 72.2 | <0.001 |
| Hossain FM et al 2022 | 1.04 | 0.92-1.17 | 0.508 | 74.2 | <0.001 |
| Klintman M et al 2022 | 1.05 | 0.93-1.17 | 0.446 | 74.1 | <0.001 |

Abbreviations: OR, odds ratio; CI, confidence intervals.

**Supplementary Figure 1:** Forest plot displaying the pooled odds ratios for the association between overweight (a) and obesity (b) and the risk of HER2-positive breast cancer, compared to underweight/normal weight, in the overall population.

Solid vertical line: Significance line (line of no effect). Gray squares: represents the weighted mean (point estimate) of each study. Horizontal bars: 95% CI line. Diamond: represents the mean of effect sizes obtained by the meta- analysis. Its width corresponds to its 95% CI.

Abbreviations: OR, odds ratio; CI, confidence intervals;

# **Supplementary Table 7**: Sensitivity analysis for the association between overweight and the risk of HER2-positive breast cancer, compared to underweight/normal weight, in the overall population

| **Study excluded** | **Random effect** | | | **I-squared (%)** | **I-sq. P-value** |
| --- | --- | --- | --- | --- | --- |
|  | **OR** | **95% CI** | **P-value** |  |  |
| Cerne JZ et al 2012 | 1.08 | 1.02-1.15 | 0.012 | 0.0 | 0.474 |
| Phipps AI et al 2012 | 1.09 | 1.02-1.16 | 0.010 | 0.0 | 0.458 |
| Horn J et al 2014 | 1.09 | 1.02-1.16 | 0.009 | 0.0 | 0.467 |
| Neuhouser ML et al 2015 | 1.07 | 1.00-1.14 | 0.043 | 0.0 | 0.895 |
| Ma H et al 2018 | 1.09 | 1.02-1.16 | 0.008 | 0.0 | 0.507 |
| Jeong SH et al 2019 | 1.11 | 0.96-1.28 | 0.151 | 0.0 | 0.466 |
| Akinyemiju T et al 2021 | 1.09 | 1.02-1.16 | 0.008 | 0.0 | 0.636 |
| Gomes KAL et al 2022 | 1.09 | 1.02-1.16 | 0.009 | 0.0 | 0.480 |
| Hossain FM et al 2022 | 1.09 | 1.02-1.16 | 0.008 | 0.0 | 0.510 |
| Klintman M et al 2022 | 1.08 | 1.02-1.15 | 0.014 | 0.0 | 0.506 |

Abbreviations: OR, odds ratio; CI, confidence intervals.

# **Supplementary Table 8**: Sensitivity analysis for the association between obesity and the risk of HER2-positive breast cancer, compared to underweight/normal weight, in the overall population

| **Study excluded** | **Random effect** | | | **I-squared (%)** | **I-sq. P-value** |
| --- | --- | --- | --- | --- | --- |
|  | **OR** | **95% CI** | **P-value** |  |  |
| Cerne JZ et al 2012 | 0.99 | 0.81-1.21 | 0.897 | 57.6 | 0.016 |
| Phipps AI et al 2012 | 1.07 | 0.88-1.31 | 0.489 | 54.9 | 0.023 |
| Horn J et al 2014 | 0.98 | 0.80-1.20 | 0.855 | 57.3 | 0.016 |
| Neuhouser ML et al 2015 | 0.99 | 0.78-1.26 | 0.929 | 60.6 | 0.009 |
| Ma H et al 2018 | 1.10 | 0.92-1.32 | 0.313 | 43.5 | 0.078 |
| Jeong SH et al 2019 | 1.01 | 0.77-1.32 | 0.972 | 61.3 | 0.008 |
| Akinyemiju T et al 2021 | 1.07 | 0.88-1.29 | 0.526 | 55.1 | 0.023 |
| Gomes KAL et al 2022 | 1.02 | 0.83-1.26 | 0.843 | 62.9 | 0.006 |
| Hossain FM et al 2022 | 1.03 | 0.81-1.30 | 0.818 | 62.5 | 0.006 |
| Klintman M et al 2022 | 1.02 | 0.82-1.26 | 0.875 | 62.9 | 0.006 |

Abbreviations: OR, odds ratio; CI, confidence intervals.

# **Supplementary Figure 2**: Forest plot displaying the pooled odds ratios for the association between overweight (a) and obesity (b) and the risk of TNBC, compared to underweight/normal weight, in the overall population.

Solid vertical line: Significance line (line of no effect). Gray squares: represents the weighted mean (point estimate) of each study. Horizontal bars: 95% CI line. Diamond: represents the mean of effect sizes obtained by the meta- analysis. Its width corresponds to its 95% CI.

Abbreviations: OR, odds ratio; CI, confidence intervals;

# **Supplementary Table 9**: Sensitivity analysis for the association between overweight and the risk of TNBC, compared to underweight/normal weight, in the overall population

| **Study excluded** | **Random effect** | | | **I-squared (%)** | **I-sq. P-value** |
| --- | --- | --- | --- | --- | --- |
|  | **OR** | **95% CI** | **P-value** |  |  |
| Millikan RC et al 2007 | 1.07 | 0.92-1.25 | 0.404 | 67.1 | <0.001 |
| Phipps AI et al 2011 | 1.06 | 0.90-1.25 | 0.473 | 67.4 | <0.001 |
| Phipps AI et al 2012 | 1.05 | 0.89-1.23 | 0.599 | 68.2 | <0.001 |
| Horn J et al 2014 | 1.07 | 0.92-1.25 | 0.366 | 67.0 | <0.001 |
| Bandera EV et al 2015 | 1.08 | 0.92-1.26 | 0.343 | 64.1 | 0.001 |
| Nagrani R et al 2016 | 1.09 | 0.94-1.26 | 0.247 | 59.0 | 0.005 |
| Ma H et al 2018 | 1.07 | 0.91-1.26 | 0.410 | 65.0 | 0.001 |
| Jeong SH et al 2019 | 1.01 | 0.91-1.11 | 0.870 | 0.0 | 0.489 |
| Akinyemiju T et al 2021 | 1.09 | 0.94-1.25 | 0.261 | 63.5 | 0.002 |
| Friebel-Klingner TM et al 2021 | 1.05 | 0.91-1.22 | 0.489 | 67.7 | <0.001 |
| Gomes KAL et al 2022 | 1.05 | 0.91-1.22 | 0.520 | 66.9 | <0.001 |
| Hossain FM et al 2022 | 1.08 | 0.93-1.26 | 0.335 | 65.3 | 0.001 |
| Klintman M et al 2022 | 1.06 | 0.91-1.23 | 0.488 | 68.2 | <0.001 |

Abbreviations: OR, odds ratio; CI, confidence intervals.

# **Supplementary Table 10**: Sensitivity analysis for the association between obesity and the risk of TNBC, compared to underweight/normal weight, in the overall population

| **Study excluded** | **Random effect** | | | **I-squared (%)** | **I-sq. P-value** |
| --- | --- | --- | --- | --- | --- |
|  | **OR** | **95% CI** | **P-value** |  |  |
| Millikan RC et al 2007 | 1.08 | 0.83-1.40 | 0.580 | 79.9 | <0.001 |
| Phipps AI et al 2011 | 1.02 | 0.78-1.34 | 0.864 | 80.0 | <0.001 |
| Phipps AI et al 2012 | 1.03 | 0.79-1.36 | 0.811 | 80.5 | <0.001 |
| Horn J et al 2014 | 1.08 | 0.84-1.39 | 0.567 | 80.3 | <0.001 |
| Bandera EV et al 2015 | 1.02 | 0.78-1.35 | 0.865 | 79.6 | <0.001 |
| Nagrani R et al 2016 | 1.10 | 0.86-1.41 | 0.461 | 78.0 | <0.001 |
| Ma H et al 2018 | 1.11 | 0.87-1.41 | 0.396 | 73.3 | <0.001 |
| Jeong SH et al 2019 | 0.98 | 0.77-1.25 | 0.878 | 75.0 | <0.001 |
| Akinyemiju T et al 2021 | 1.09 | 0.85-1.39 | 0.504 | 79.7 | <0.001 |
| Friebel-Klingner TM et al 2021 | 1.03 | 0.80-1.33 | 0.807 | 80.6 | <0.001 |
| Gomes KAL et al 2022 | 0.98 | 0.78-1.24 | 0.894 | 76.5 | <0.001 |
| Hossain FM et al 2022 | 1.04 | 0.79-1.37 | 0.773 | 80.8 | <0.001 |
| Klintman M et al 2022 | 1.07 | 0.83-1.37 | 0.618 | 80.5 | <0.001 |

Abbreviations: OR, odds ratio; CI, confidence intervals.

# **Supplementary Table 11**: Sensitivity analysis for the association between overweight and the risk of ER-positive breast cancer, compared to underweight/normal weight, in postmenopausal women

| **Study excluded** | **Random effect** | | | **I-squared (%)** | **I-sq. P-value** |
| --- | --- | --- | --- | --- | --- |
|  | **OR** | **95% CI** | **P-value** |  |  |
| Li CI et al 2006 | 1.13 | 1.06-1.22 | <0.001 | 69.2 | <0.001 |
| Rosenberg LU et al 2006 | 1.13 | 1.06-1.21 | 0.001 | 68.9 | <0.001 |
| Chlebowski RT et al 2007 | 1.14 | 1.06-1.22 | 0.001 | 69.5 | <0.001 |
| Millikan RC et al 2007 | 1.15 | 1.08-1.23 | <0.001 | 65.4 | <0.001 |
| Berstard P et al 2010 | 1.16 | 1.08-1.23 | <0.001 | 64.6 | <0.001 |
| Barnes B et al 2011 | 1.15 | 1.08-1.23 | <0.001 | 65.1 | <0.001 |
| Phipps AI et al 2011 | 1.14 | 1.06-1.23 | <0.001 | 68.2 | <0.001 |
| Cerne JZ et al 2012 | 1.13 | 1.05-1.21 | 0.001 | 68.4 | <0.001 |
| Phipps AI et al 2012 | 1.14 | 1.05-1.23 | 0.001 | 69.5 | <0.001 |
| Kawai M et al 2013 | 1.13 | 1.05-1.21 | 0.001 | 68.4 | <0.001 |
| Horn J et al 2014 | 1.14 | 1.06-1.22 | <0.001 | 69.4 | <0.001 |
| Robinson W et al 2014 | 1.14 | 1.07-1.22 | <0.001 | 69.3 | <0.001 |
| Bandera EV et al 2015 | 1.14 | 1.06-1.22 | <0.001 | 69.4 | <0.001 |
| Neuhouser ML et al 2015 | 1.12 | 1.05-1.19 | 0.001 | 59.2 | <0.001 |
| John EM et al 2016 | 1.14 | 1.07-1.23 | <0.001 | 69.2 | <0.001 |
| Nagrani R et al 2016 | 1.13 | 1.06-1.21 | <0.001 | 68.5 | <0.001 |
| McClain KM et al 2017 | 1.13 | 1.05-1.21 | 0.001 | 68.2 | <0.001 |
| Wang F et al 2017 | 1.14 | 1.07-1.23 | <0.001 | 68.7 | <0.001 |
| Ma H et al 2018 | 1.15 | 1.08-1.23 | <0.001 | 67.1 | <0.001 |
| Dashti GS et al 2019 | 1.14 | 1.06-1.22 | <0.001 | 69.4 | <0.001 |
| Jeong SH et al 2019 | 1.13 | 1.05-1.21 | 0.001 | 66.2 | <0.001 |
| Klintman M et al 2022 | 1.13 | 1.06-1.22 | 0.001 | 69.3 | <0.001 |

Abbreviations: OR, odds ratio; CI, confidence intervals.

# **Supplementary Table 12**: Sensitivity analysis for the association between obesity and the risk of ER-positive breast cancer, compared to underweight/normal weight, in postmenopausal women

| **Study excluded** | **Random effect** | | | **I-squared (%)** | **I-sq. P-value** |
| --- | --- | --- | --- | --- | --- |
|  | **OR** | **95% CI** | **P-value** |  |  |
| Li CI et al 2006 | 1.30 | 1.18-1.42 | <0.001 | 75.3 | <0.001 |
| Rosenberg LU et al 2006 | 1.28 | 1.17-1.40 | <0.001 | 73.3 | <0.001 |
| Chlebowski RT et al 2007 | 1.30 | 1.18-1.43 | <0.001 | 75.3 | <0.001 |
| Millikan RC et al 2007 | 1.32 | 1.21-1.44 | <0.001 | 72.2 | <0.001 |
| Berstard P et al 2010 | 1.31 | 1.20-1.44 | <0.001 | 72.8 | <0.001 |
| Barnes B et al 2011 | 1.32 | 1.21-1.44 | <0.001 | 71.4 | <0.001 |
| Phipps AI et al 2011 | 1.31 | 1.18-1.44 | <0.001 | 74.5 | <0.001 |
| Cerne JZ et al 2012 | 1.27 | 1.16-1.39 | <0.001 | 73.2 | <0.001 |
| Phipps AI et al 2012 | 1.30 | 1.17-1.44 | <0.001 | 75.2 | <0.001 |
| Kawai M et al 2013 | 1.25 | 1.16-1.35 | <0.001 | 62.3 | <0.001 |
| Horn J et al 2014 | 1.29 | 1.17-1.41 | <0.001 | 75.1 | <0.001 |
| Robinson W et al 2014 | 1.29 | 1.18-1.42 | <0.001 | 75.3 | <0.001 |
| Bandera EV et al 2015 | 1.30 | 1.18-1.43 | <0.001 | 75.2 | <0.001 |
| Neuhouser ML et al 2015 | 1.30 | 1.17-1.43 | <0.001 | 75.1 | <0.001 |
| John EM et al 2016 | 1.31 | 1.19-1.43 | <0.001 | 74.6 | <0.001 |
| Nagrani R et al 2016 | 1.29 | 1.18-1.42 | <0.001 | 75.3 | <0.001 |
| McClain KM et al 2017 | 1.28 | 1.17-1.40 | <0.001 | 73.9 | <0.001 |
| Wang F et al 2017 | 1.30 | 1.19-1.42 | <0.001 | 75.1 | <0.001 |
| Ma H et al 2018 | 1.30 | 1.18-1.42 | <0.001 | 75.2 | <0.001 |
| Dashti GS et al 2019 | 1.28 | 1.17-1.40 | <0.001 | 74.6 | <0.001 |
| Jeong SH et al 2019 | 1.28 | 1.17-1.41 | <0.001 | 74.8 | <0.001 |
| Klintman M et al 2022 | 1.30 | 1.18-1.42 | <0.001 | 75.3 | <0.001 |

Abbreviations: OR, odds ratio; CI, confidence intervals.

# **Supplementary Figure 3**: Forest plot displaying the pooled odds ratios for the association between overweight (a) and obesity (b) and the risk of ER-negative breast cancer, compared to underweight/normal weight, in the postmenopausal women

 Solid vertical line: Significance line (line of no effect). Gray squares: represents the weighted mean (point estimate) of each study. Horizontal bars: 95% CI line. Diamond: represents the mean of effect sizes obtained by the meta- analysis. Its width corresponds to its 95% CI.

Abbreviations: OR, odds ratio; CI, confidence intervals;

# **Supplementary Table 13**: Sensitivity analysis for the association between overweight and the risk of ER-negative breast cancer, compared to underweight/normal weight, in postmenopausal women

| **Study excluded** | **Random effect** | | | **I-squared (%)** | **I-sq. P-value** |
| --- | --- | --- | --- | --- | --- |
|  | **OR** | **95% CI** | **P-value** |  |  |
| Li CI et al 2006 | 1.04 | 0.97-1.11 | 0.313 | 0.0 | 0.634 |
| Rosenberg LU et al 2006 | 1.03 | 0.96-1.11 | 0.365 | 0.0 | 0.656 |
| Chlebowski RT et al 2007 | 1.05 | 0.98-1.13 | 0.150 | 0.0 | 0.807 |
| Millikan RC et al 2007 | 1.04 | 0.97-1.12 | 0.238 | 0.0 | 0.699 |
| Berstard P et al 2010 | 1.04 | 0.96-1.11 | 0.343 | 0.0 | 0.634 |
| Barnes B et al 2011 | 1.04 | 0.97-1.12 | 0.255 | 0.0 | 0.643 |
| Phipps AI et al 2011 | 1.04 | 0.96-1.11 | 0.338 | 0.0 | 0.635 |
| Cerne JZ et al 2012 | 1.04 | 0.97-1.11 | 0.313 | 0.0 | 0.635 |
| Phipps AI et al 2012 | 1.04 | 0.97-1.12 | 0.292 | 0.0 | 0.631 |
| Kawai M et al 2013 | 1.05 | 0.97-1.12 | 0.219 | 0.0 | 0.717 |
| Horn J et al 2014 | 1.04 | 0.97-1.12 | 0.257 | 0.0 | 0.653 |
| Robinson W et al 2014 | 1.04 | 0.97-1.11 | 0.325 | 0.0 | 0.642 |
| Bandera EV et al 2015 | 1.06 | 0.98-1.14 | 0.146 | 0.0 | 0.783 |
| Neuhouser ML et al 2015 | 1.02 | 0.95-1.09 | 0.637 | 0.0 | 0.865 |
| John EM et al 2016 | 1.04 | 0.97-1.12 | 0.236 | 0.0 | 0.669 |
| Nagrani R et al 2016 | 1.03 | 0.96-1.11 | 0.402 | 0.0 | 0.664 |
| Wang F et al 2017 | 1.04 | 0.97-1.12 | 0.255 | 0.0 | 0.658 |
| Ma H et al 2018 | 1.03 | 0.96-1.11 | 0.356 | 0.0 | 0.646 |
| Jeong SH et al 2019 | 1.02 | 0.95-1.10 | 0.623 | 0.0 | 0.782 |
| Klintman M et al 2022 | 1.04 | 0.97-1.11 | 0.285 | 0.0 | 0.632 |

Abbreviations: OR, odds ratio; CI, confidence intervals.

# **Supplementary Table 14**: Sensitivity analysis for the association between obesity and the risk of ER-negative breast cancer, compared to underweight/normal weight, in postmenopausal women

| **Study excluded** | **Random effect** | | | **I-squared (%)** | **I-sq. P-value** |
| --- | --- | --- | --- | --- | --- |
|  | **OR** | **95% CI** | **P-value** |  |  |
| Li CI et al 2006 | 1.03 | 0.90-1.17 | 0.683 | 47.9 | 0.011 |
| Rosenberg LU et al 2006 | 1.00 | 0.88-1.14 | 0.989 | 42.6 | 0.026 |
| Chlebowski RT et al 2007 | 1.01 | 0.88-1.15 | 0.898 | 45.6 | 0.016 |
| Millikan RC et al 2007 | 1.04 | 0.92-1.17 | 0.559 | 44.0 | 0.021 |
| Berstard P et al 2010 | 1.05 | 0.92-1.19 | 0.480 | 41.4 | 0.031 |
| Barnes B et al 2011 | 1.02 | 0.90-1.17 | 0.724 | 48.2 | 0.010 |
| Phipps AI et al 2011 | 1.00 | 0.88-1.14 | 0.968 | 43.3 | 0.024 |
| Cerne JZ et al 2012 | 1.00 | 0.89-1.11 | 0.926 | 31.5 | 0.094 |
| Phipps AI et al 2012 | 1.03 | 0.90-1.18 | 0.650 | 47.4 | 0.012 |
| Kawai M et al 2013 | 1.01 | 0.89-1.14 | 0.878 | 44.7 | 0.019 |
| Horn J et al 2014 | 1.02 | 0.90-1.17 | 0.729 | 48.2 | 0.010 |
| Robinson W et al 2014 | 1.01 | 0.89-1.15 | 0.880 | 46.3 | 0.015 |
| Bandera EV et al 2015 | 1.03 | 0.91-1.18 | 0.623 | 46.7 | 0.013 |
| Neuhouser ML et al 2015 | 1.03 | 0.90-1.18 | 0.692 | 48.0 | 0.011 |
| John EM et al 2016 | 1.04 | 0.92-1.18 | 0.509 | 42.6 | 0.026 |
| Nagrani R et al 2016 | 1.04 | 0.91-1.18 | 0.572 | 45.6 | 0.016 |
| Wang F et al 2017 | 1.03 | 0.90-1.17 | 0.693 | 48.0 | 0.011 |
| Ma H et al 2018 | 1.03 | 0.90-1.18 | 0.649 | 47.5 | 0.012 |
| Jeong SH et al 2019 | 1.01 | 0.89-1.14 | 0.889 | 45.1 | 0.018 |
| Klintman M et al 2022 | 1.03 | 0.91-1.17 | 0.671 | 47.5 | 0.012 |

Abbreviations: OR, odds ratio; CI, confidence intervals.

# **Supplementary Figure 4**: Forest plot displaying the pooled odds ratios for the association between overweight (a) and obesity (b) and the risk of HER2-positive BC, compared to underweight/normal weight, in the postmenopausal women

Solid vertical line: Significance line (line of no effect). Gray squares: represents the weighted mean (point estimate) of each study. Horizontal bars: 95% CI line. Diamond: represents the mean of effect sizes obtained by the meta- analysis. Its width corresponds to its 95% CI.

Abbreviations: OR, odds ratio; CI, confidence intervals;

# **Supplementary Table 15**: Sensitivity analysis for the association between overweight and the risk of HER2-positive breast cancer, compared to underweight/normal weight, in postmenopausal women

| **Study excluded** | **Random effect** | | | **I-squared (%)** | **I-sq. P-value** |
| --- | --- | --- | --- | --- | --- |
|  | **OR** | **95% CI** | **P-value** |  |  |
| Cerne JZ et al 2012 | 1.13 | 1.05-1.22 | 0.002 | 0.0 | 0.560 |
| Phipps AI et al 2012 | 1.13 | 1.05-1.23 | 0.002 | 0.0 | 0.547 |
| Horn J et al 2014 | 1.14 | 1.05-1.23 | 0.002 | 0.0 | 0.583 |
| Neuhouser ML et al 2015 | 1.11 | 1.02-1.20 | 0.013 | 0.0 | 0.973 |
| Ma H et al 2018 | 1.13 | 1.04-1.22 | 0.002 | 0.0 | 0.560 |
| Jeong SH et al 2019 | 1.28 | 1.07-1.53 | 0.007 | 0.0 | 0.870 |
| Klintman M et al 2022 | 1.13 | 1.04-1.22 | 0.003 | 0.0 | 0.591 |

Abbreviations: OR, odds ratio; CI, confidence intervals.

| **Study excluded** | **Random effect** | | | **I-squared (%)** | **I-sq. P-value** |
| --- | --- | --- | --- | --- | --- |
|  | **OR** | **95% CI** | **P-value** |  |  |
| Cerne JZ et al 2012 | 1.16 | 0.98-1.37 | 0.096 | 22.5 | 0.265 |
| Phipps AI et al 2012 | 1.20 | 1.07-1.35 | 0.003 | 0.0 | 0.548 |
| Horn J et al 2014 | 1.15 | 0.97-1.37 | 0.118 | 22.9 | 0.262 |
| Neuhouser ML et al 2015 | 1.19 | 0.91-1.55 | 0.214 | 39.2 | 0.144 |
| Ma H et al 2018 | 1.20 | 0.97-1.48 | 0.088 | 40.7 | 0.134 |
| Jeong SH et al 2019 | 1.22 | 0.92-1.63 | 0.170 | 38.1 | 0.152 |
| Klintman M et al 2022 | 1.20 | 0.97-1.47 | 0.093 | 40.8 | 0.133 |

# **Supplementary Table 16**: Sensitivity analysis for the association between obesity and the risk of HER2-positive breast cancer, compared to underweight/normal weight, in postmenopausal women

Abbreviations: OR, odds ratio; CI, confidence intervals.

# **Supplementary Figure 5**: Forest plot displaying the pooled odds ratios for the association between overweight (a) and obesity (b) and the risk of TNBC, compared to underweight/normal weight, in the postmenopausal women

Solid vertical line: Significance line (line of no effect). Gray squares: represents the weighted mean (point estimate) of each study. Horizontal bars: 95% CI line. Diamond: represents the mean of effect sizes obtained by the meta- analysis. Its width corresponds to its 95% CI.

Abbreviations: OR, odds ratio; CI, confidence intervals;

# **Supplementary Table 17**: Sensitivity analysis for the association between overweight and the risk of TNBC, compared to underweight/normal weight, in postmenopausal women

| **Study excluded** | **Random effect** | | | **I-squared (%)** | **I-sq. P-value** |
| --- | --- | --- | --- | --- | --- |
|  | **OR** | **95% CI** | **P-value** |  |  |
| Millikan RC et al 2007 | 1.07 | 0.90-1.28 | 0.451 | 61.6 | 0.011 |
| Phipps AI et al 2011 | 1.04 | 0.85-1.27 | 0.734 | 65.2 | 0.005 |
| Phipps AI et al 2012 | 1.06 | 0.87-1.28 | 0.583 | 63.0 | 0.008 |
| Horn J et al 2014 | 1.06 | 0.88-1.27 | 0.554 | 64.3 | 0.007 |
| Bandera EV et al 2015 | 1.15 | 1.01-1.32 | 0.034 | 28.7 | 0.199 |
| Nagrani R et al 2016 | 1.02 | 0.83-1.24 | 0.881 | 65.9 | 0.004 |
| Ma H et al 2018 | 1.04 | 0.85-1.26 | 0.729 | 65.8 | 0.005 |
| Jeong SH et al 2019 | 0.99 | 0.85-1.15 | 0.909 | 20.4 | 0.267 |
| Klintman M et al 2022 | 1.03 | 0.86-1.25 | 0.734 | 66.0 | 0.004 |

Abbreviations: OR, odds ratio; CI, confidence intervals.

# **Supplementary Table 18**: Sensitivity analysis for the association between obesity and the risk of TNBC, compared to underweight/normal weight, in postmenopausal women

| **Study excluded** | **Random effect** | | | **I-squared (%)** | **I-sq. P-value** |
| --- | --- | --- | --- | --- | --- |
|  | **OR** | **95% CI** | **P-value** |  |  |
| Millikan RC et al 2007 | 1.07 | 0.76-1.52 | 0.692 | 84.4 | <0.001 |
| Phipps AI et al 2011 | 0.96 | 0.64-1.45 | 0.848 | 86.3 | <0.001 |
| Phipps AI et al 2012 | 1.00 | 0.68-1.47 | 0.986 | 85.6 | <0.001 |
| Horn J et al 2014 | 1.05 | 0.74-1.50 | 0.771 | 85.1 | <0.001 |
| Bandera EV et al 2015 | 1.02 | 0.71-1.49 | 0.902 | 84.8 | <0.001 |
| Nagrani R et al 2016 | 1.03 | 0.72-1.49 | 0.866 | 85.0 | <0.001 |
| Ma H et al 2018 | 1.02 | 0.70-1.47 | 0.932 | 85.7 | <0.001 |
| Jeong SH et al 2019 | 0.97 | 0.82-1.15 | 0.731 | 15.6 | 0.307 |
| Klintman M et al 2022 | 1.04 | 0.73-1.47 | 0.826 | 85.8 | <0.001 |

Abbreviations: OR, odds ratio; CI, confidence intervals.

# **Supplementary Table 19**: Sensitivity analysis for the association between overweight and the ER-positive, compared to underweight/normal weight, in premenopausal women

| **Study excluded** | **Random effect** | | | **I-squared (%)** | **I-sq. P-value** |
| --- | --- | --- | --- | --- | --- |
|  | **OR** | **95% CI** | **P-value** |  |  |
| Ma H et al 2006 | 0.78 | 0.69-0.89 | <0.001 | 73.1 | <0.001 |
| Millikan RC et al 2007 | 0.81 | 0.71-0.93 | 0.002 | 74.5 | <0.001 |
| Berstard P et al 2010 | 0.80 | 0.70-0.92 | 0.002 | 75.2 | <0.001 |
| Phipps AI et al 2012 | 0.81 | 0.70-0.93 | 0.003 | 73.3 | <0.001 |
| Kawai M et al 2013 | 0.80 | 0.70-0.91 | 0.001 | 75.1 | <0.001 |
| Robinson W et al 2014 | 0.81 | 0.72-0.93 | 0.002 | 73.9 | <0.001 |
| Bandera EV et al 2015 | 0.79 | 0.69-0.90 | 0.001 | 74.4 | <0.001 |
| John EM et al 2015 | 0.82 | 0.71-0.93 | 0.002 | 73.6 | <0.001 |
| Nagrani R et al 2016 | 0.81 | 0.71-0.93 | 0.002 | 74.4 | <0.001 |
| Wang F et al 2017 | 0.84 | 0.75-0.94 | 0.002 | 62.7 | 0.003 |
| Ma H et al 2018 | 0.80 | 0.70-0.92 | 0.002 | 75.2 | <0.001 |
| Jeong SH et al 2019 | 0.78 | 0.70-0.87 | <0.001 | 51.9 | 0.023 |

Abbreviations: OR, odds ratio; CI, confidence intervals.

# **Supplementary Table 20**: Sensitivity analysis for the association between obesity and the ER-positive, compared to underweight/normal weight, in premenopausal women

| **Study excluded** | **Random effect** | | | **I-squared (%)** | **I-sq. P-value** |
| --- | --- | --- | --- | --- | --- |
|  | **OR** | **95% CI** | **P-value** |  |  |
| Ma H et al 2006 | 0.88 | 0.75-1.03 | 0.106 | 76.3 | <0.001 |
| Millikan RC et al 2007 | 0.89 | 0.77-1.04 | 0.156 | 75.2 | <0.001 |
| Berstard P et al 2010 | 0.89 | 0.76-1.04 | 0.147 | 75.6 | <0.001 |
| Phipps AI et al 2012 | 0.88 | 0.74-1.04 | 0.136 | 76.2 | <0.001 |
| Kawai M et al 2013 | 0.89 | 0.76-1.03 | 0.114 | 76.0 | <0.001 |
| Robinson W et al 2014 | 0.90 | 0.77-1.05 | 0.184 | 73.6 | <0.001 |
| Bandera EV et al 2015 | 0.85 | 0.73-1.00 | 0.046 | 73.7 | <0.001 |
| John EM et al 2015 | 0.88 | 0.74-1.04 | 0.136 | 76.2 | <0.001 |
| Nagrani R et al 2016 | 0.91 | 0.79-1.05 | 0.187 | 71.4 | <0.001 |
| Wang F et al 2017 | 0.84 | 0.75-0.94 | 0.002 | 52.5 | 0.021 |
| Ma H et al 2018 | 0.88 | 0.75-1.04 | 0.133 | 76.0 | <0.001 |
| Jeong SH et al 2019 | 0.86 | 0.73-1.01 | 0.067 | 74.5 | <0.001 |

Abbreviations: OR, odds ratio; CI, confidence intervals.

# **Supplementary Figure 6**: Forest plot displaying the pooled odds ratios for the association between overweight (a) and obesity (b) and the risk of ER-negative breast cancer, compared to underweight/normal weight, in the premenopausal women

Solid vertical line: Significance line (line of no effect). Gray squares: represents the weighted mean (point estimate) of each study. Horizontal bars: 95% CI line. Diamond: represents the mean of effect sizes obtained by the meta- analysis. Its width corresponds to its 95% CI.

Abbreviations: OR, odds ratio; CI, confidence intervals;

# **Supplementary Table 21**: Sensitivity analysis for the association between overweight and the ER-negative, compared to underweight/normal weight, in premenopausal women

| **Study excluded** | **Random effect** | | | **I-squared (%)** | **I-sq. P-value** |
| --- | --- | --- | --- | --- | --- |
|  | **OR** | **95% CI** | **P-value** |  |  |
| Ma H et al 2006 | 1.14 | 1.04-1.25 | 0.007 | 0.0 | 0.785 |
| Millikan RC et al 2007 | 1.15 | 1.05-1.26 | 0.002 | 0.0 | 0.665 |
| Berstard P et al 2010 | 1.14 | 1.03-1.26 | 0.009 | 0.0 | 0.697 |
| Phipps AI et al 2012 | 1.14 | 1.04-1.25 | 0.005 | 0.0 | 0.733 |
| Kawai M et al 2013 | 1.15 | 1.05-1.26 | 0.002 | 0.0 | 0.664 |
| Robinson W et al 2014 | 1.17 | 1.06-1.28 | 0.001 | 0.0 | 0.747 |
| Bandera EV et al 2015 | 1.15 | 1.04-1.26 | 0.004 | 0.0 | 0.665 |
| John EM et al 2015 | 1.17 | 1.06-1.28 | 0.001 | 0.0 | 0.744 |
| Nagrani R et al 2016 | 1.18 | 1.07-1.29 | 0.001 | 0.0 | 0.852 |
| Wang F et al 2017 | 1.15 | 1.05-1.26 | 0.004 | 0.0 | 0.679 |
| Ma H et al 2018 | 1.17 | 1.07-1.29 | 0.001 | 0.0 | 0.760 |
| Jeong SH et al 2019 | 1.13 | 1.03-1.25 | 0.014 | 0.0 | 0.738 |

Abbreviations: OR, odds ratio; CI, confidence intervals.

# **Supplementary Table 22**: Sensitivity analysis for the association between obesity and the ER-negative, compared to underweight/normal weight, in premenopausal women

| **Study excluded** | **Random effect** | | | **I-squared (%)** | **I-sq. P-value** |
| --- | --- | --- | --- | --- | --- |
|  | **OR** | **95% CI** | **P-value** |  |  |
| Ma H et al 2006 | 1.06 | 0.92-1.24 | 0.421 | 38.9 | 0.089 |
| Millikan RC et al 2007 | 1.11 | 0.94-1.31 | 0.208 | 52.0 | 0.022 |
| Berstard P et al 2010 | 1.12 | 0.94-1.33 | 0.204 | 51.0 | 0.026 |
| Phipps AI et al 2012 | 1.10 | 0.93-1.31 | 0.259 | 52.3 | 0.021 |
| Kawai M et al 2013 | 1.11 | 0.94-1.30 | 0.208 | 52.2 | 0.022 |
| Robinson W et al 2014 | 1.12 | 0.95-1.33 | 0.188 | 50.8 | 0.026 |
| Bandera EV et al 2015 | 1.11 | 0.93-1.32 | 0.250 | 52.3 | 0.021 |
| John EM et al 2015 | 1.12 | 0.94-1.33 | 0.194 | 51.2 | 0.025 |
| Nagrani R et al 2016 | 1.16 | 1.02-1.31 | 0.025 | 19.5 | 0.258 |
| Wang F et al 2017 | 1.07 | 0.93-1.24 | 0.355 | 37.6 | 0.099 |
| Ma H et al 2018 | 1.11 | 0.93-1.32 | 0.249 | 52.3 | 0.021 |
| Jeong SH et al 2019 | 1.08 | 0.91-1.27 | 0.384 | 46.4 | 0.045 |

Abbreviations: OR, odds ratio; CI, confidence intervals.

# **Supplementary Figure 7**: Forest plot displaying the pooled odds ratios for the association between overweight (a) and obesity (b) and the risk of HER2-positive breast cancer, compared to underweight/normal weight, in the premenopausal women

Solid vertical line: Significance line (line of no effect). Gray squares: represents the weighted mean (point estimate) of each study. Horizontal bars: 95% CI line. Diamond: represents the mean of effect sizes obtained by the meta- analysis. Its width corresponds to its 95% CI.

Abbreviations: OR, odds ratio; CI, confidence intervals;

# **Supplementary Table 23**: Sensitivity analysis for the association between overweight and the risk of HER2-positive, compared to underweight/normal weight, in premenopausal women

| **Study excluded** | **Random effect** | | | **I-squared (%)** | **I-sq. P-value** |
| --- | --- | --- | --- | --- | --- |
|  | **OR** | **95% CI** | **P-value** |  |  |
| Phipps AI et al 2012 | 1.03 | 0.92-1.15 | 0.637 | 0.0 | 0.876 |
| Ma H et al 2018 | 1.02 | 0.91-1.15 | 0.701 | 0.0 | 0.424 |
| Jeong SH et al 2019 | 0.92 | 0.61-1.39 | 0.679 | 0.0 | 0.542 |

Abbreviations: OR, odds ratio; CI, confidence intervals.

**Supplementary Table 24**: Sensitivity analysis for the association between overweight and the risk of HER2-positive, compared to underweight/normal weight, in premenopausal women

| **Study excluded** | **Random effect** | | | **I-squared (%)** | **I-sq. P-value** |
| --- | --- | --- | --- | --- | --- |
|  | **OR** | **95% CI** | **P-value** |  |  |
| Phipps AI et al 2012 | 1.11 | 0.90-1.37 | 0.325 | 0.0 | 0.495 |
| Ma H et al 2018 | 1.06 | 0.85-1.31 | 0.628 | 0.0 | 0.442 |
| Jeong SH et al 2019 | 1.11 | 0.66-1.85 | 0.705 | 13.0 | 0.284 |

Abbreviations: OR, odds ratio; CI, confidence intervals.

# **Supplementary Figure 8**: Forest plot displaying the pooled odds ratios for the association between overweight (a) and obesity (b) and the risk of TNBC, compared to underweight/normal weight, in the premenopausal women

Solid vertical line: Significance line (line of no effect). Gray squares: represents the weighted mean (point estimate) of each study. Horizontal bars: 95% CI line. Diamond: represents the mean of effect sizes obtained by the meta- analysis. Its width corresponds to its 95% CI.

Abbreviations: OR, odds ratio; CI, confidence intervals;

# **Supplementary Table 25**: Sensitivity analysis for the association between overweight and the risk of TNBC, compared to underweight/normal weight, in premenopausal women

| **Study excluded** | **Random effect** | | | **I-squared (%)** | **I-sq. P-value** |
| --- | --- | --- | --- | --- | --- |
|  | **OR** | **95% CI** | **P-value** |  |  |
| Millikan RC et al 2007 | 1.30 | 1.13-1.50 | <0.001 | 25.2 | 0.254 |
| Phipps AI et al 2012 | 1.28 | 1.14-1.45 | <0.001 | 12.4 | 0.335 |
| Bandera EV et al 2015 | 1.27 | 1.08-1.49 | 0.004 | 32.2 | 0.207 |
| Nagrani R et al 2016 | 1.35 | 1.23-1.49 | <0.001 | 1.2 | 0.400 |
| Ma H et al 2018 | 1.36 | 1.23-1.49 | <0.001 | 0.0 | 0.413 |
| Jeong SH et al 2019 | 1.20 | 1.01-1.44 | 0.044 | 3.0 | 0.390 |

Abbreviations: OR, odds ratio; CI, confidence intervals.

# **Supplementary Table 26:** Sensitivity analysis for the association between obesity and the risk of TNBC, compared to underweight/normal weight, in premenopausal women

| **Study excluded** | **Random effect** | | | **I-squared (%)** | **I-sq. P-value** |
| --- | --- | --- | --- | --- | --- |
|  | **OR** | **95% CI** | **P-value** |  |  |
| Millikan RC et al 2007 | 1.26 | 0.95-1.67 | 0.105 | 58.1 | 0.049 |
| Phipps AI et al 2012 | 1.19 | 0.88-1.61 | 0.249 | 62.6 | 0.030 |
| Bandera EV et al 2015 | 1.16 | 0.86-1.57 | 0.342 | 60.8 | 0.037 |
| Nagrani R et al 2016 | 1.41 | 1.19-1.67 | <0.001 | 2.1 | 0.394 |
| Ma H et al 2018 | 1.24 | 0.91-1.68 | 0.166 | 59.4 | 0.043 |
| Jeong SH et al 2019 | 1.12 | 0.86-1.47 | 0.397 | 36.3 | 0.179 |

Abbreviations: OR, odds ratio; CI, confidence intervals.
